# Decision models in type 2 diabetes mellitus: A systematic review

**DOI:** 10.1007/s00592-021-01742-6

**Published:** 2021-06-03

**Authors:** Jiayu Li, Yun Bao, Xuedi Chen, Limin Tian

**Affiliations:** 1 Department of Endocrinology, Gansu Provincial Hospital, Lanzhou, 730000 Gansu Province China; 2 Clinical Research Center for Metabolic Diseases, No. 204 Donggang west road, Lanzhou, 730000 Gansu Province China; 3 School of Clinical Medicine, Ningxia Medical University, Yinchuan, 750004 Ningxia Province China

**Keywords:** Type 2 diabetes mellitus, Decision model, Simulation, Cost-utility

## Abstract

**Aims:**

To reduce the burden of type 2 diabetes (T2DM), the disease decision model plays a vital role in supporting decision-making. Currently, there is no comprehensive summary and assessment of the existing decision models for T2DM. The objective of this review is to provide an overview of the characteristics and capabilities of published decision models for T2DM. We also discuss which models are suitable for different study demands.

**Materials and methods:**

Four databases (PubMed, Web of Science, Embase, and the Cochrane Library) were electronically searched for papers published from inception to August 2020. Search terms were: “Diabetes-Mellitus, Type 2”, “cost-utility”, “quality-of-life”, and “decision model”. Reference lists of the included studies were manually searched. Two reviewers independently screened the titles and abstracts following the inclusion and exclusion criteria. If there was insufficient information to include or exclude a study, then a full-text version was sought. The extracted information included basic information, study details, population characteristics, basic modeling methodologies, model structure, and data inputs for the included applications, model outcomes, model validation, and uncertainty.

**Results:**

Fourteen unique decision models for T2DM were identified. Markov chains and risk equations were utilized by four and three models, respectively. Three models utilized both. Except for the Archimedes model, all other models (*n* = 13) implemented an annual cycle length. The time horizon of most models was flexible. Fourteen models had differences in the division of health states. Ten models emphasized macrovascular and microvascular complications. Six models included adverse events. Majority of the models (*n* = 11) were patient-level simulation models. Eleven models simulated annual changes in risk factors (body mass index, glycemia, HbA1c, blood pressure (systolic and/or diastolic), and lipids (total cholesterol and/or high-density lipoprotein)). All models reported the main data sources used to develop health states of complications. Most models (*n* = 11) could deal with the uncertainty of models, which were described in varying levels of detail in the primary studies. Eleven studies reported that one or more validation checks were performed.

**Conclusions:**

The existing decision models for T2DM are heterogeneous in terms of the level of detail in the classification of health states. Thus, more attention should be focused on balancing the desired level of complexity against the required level of transparency in the development of T2DM decision models.

**Supplementary Information:**

The online version contains supplementary material available at 10.1007/s00592-021-01742-6.

## Introduction

Diabetes is a major health issue that has reached alarming levels. Today, nearly half a billion people are living with diabetes worldwide. In 2017, it was estimated that 425 million people had diabetes (types 1 and 2 combined), increasing to 463 million in 2019, and this number is projected to reach 578 million by 2030 [1]. Due to population growth and aging, the Global Burden of Disease Study showed that all-age disability-adjusted life-years (DALYs) of people with diabetes in 2016 were 57,233.7, which increased by 24.4% from 1990 to 2016 [2]. To decrease the high disease burden [3–5], efficient prevention and treatment of diabetes and its complications are major tasks for health policy. In these situations, disease decision models play a vital role in supporting decision-making for evaluating the long-term health and economic outcomes of interventions in the public and private health sectors [6].

Disease decision models are logical mathematical frameworks that synthesize the available data (e.g., short-run clinical trial outcomes, risk equations, and progression rates) and known physiologic relationships into a coherent internally consistent framework that can be extrapolated over time [7, 8]. Many models have been developed and validated for type 2 diabetes mellitus (T2DM) populations and used in a variety of ways, such as estimating long-term clinical outcomes and costs of a clinical trial and aiding decision makers in choosing between available interventions in these populations [9–12]. For instance, the Centers for Disease Control (CDC) Diabetes Cost-effectiveness Group used the Diabetes Cost-Effectiveness Model (DCEM) to estimate the incremental cost-effectiveness of intensive glycemic control (relative to conventional control), intensified hypertension control, and reduction in serum cholesterol levels in patients with T2DM [12]. From a modeling standpoint, T2DM ranks among the most challenging disease areas because of its impact on multiple interrelated organ systems and multiple treatment goals (including blood glucose, blood pressure, and blood lipids) [13]. However, unlike models in type 1 diabetes mellitus (T1DM) and prediabetes [14, 15], there are few comprehensive summaries and assessments of the existing decision models for T2DM.

Our research provides an overview of the characteristics and capabilities of published decision models in T2DM. We also discuss which models are more suitable for different study demands.

## Methods

### Search strategy and selection criteria

This systematic review was conducted and reported in accordance with the Preferred Reporting Items for Systematic Reviews and Meta-Analyses (PRISMA) guidelines [16].

Four databases (PubMed, Web of Science, Embase, and the Cochrane Library) were electronically searched for papers that were published from inception to August 2020. The following search terms/MeSH terms were used: “Diabetes Mellitus”, “Type 2”, “cost-utility”, “quality of life”, and “decision model”. The integral search strategy is provided in Appendix 1. We also manually searched the reference lists of the included studies. References were managed using ENDNOTE X9 (Clarivate, Philadelphia, PA). Studies were eligible for inclusion if they met the following predefined criteria:

1. Population: Patients with T2DM; modeling studies conducted in a mixed population (T1DM and T2DM) were included only if the model adaptation for T2DM patients was reported separately in the full-text publication;
2. Intervention and comparators: No restrictions;
3. Outcomes: Studies with decision models in T2DM that reported health economics outcomes such as costs, (quality-adjusted) life expectancy, and diabetes-related complications;
4. Study design: All modeling studies capable of performing a full economic evaluation were included.

The exclusion criteria were as follows:

1. Population: T1DM only, or gestational diabetes or maturity-onset diabetes of the young (MODY);
2. Outcomes: Modeling studies with a limited focus on particular sub-components of T2DM (e.g., only one complication of T2DM), or modeling application studies with a time horizon of ≤ 5 years;
3. Study design: Abstracts or full-text unavailable.

Two reviewers (L.J. and C.X) independently screened the titles and abstracts according to the inclusion criteria. If there was insufficient information to include or exclude a study, then a full-text version was sought. A consensus between both reviewers was required. Full-text versions of all the relevant studies were also obtained and read by two independent reviewers (L.J. and B.Y.) to ensure that the inclusion criteria were met. Any disagreement between the two reviewers was resolved by a third reviewer for assessment. If there was insufficient information to include a study, then the authors were contacted when possible.

### Quality assessment

Two reviewers (L.J. and B.Y.) independently assessed the quality of all the included studies by using the Philips et al. [17] checklist, which assesses the quality of reporting of the decision models and model-based economic evaluations, as recommended in the Cochrane Handbook for Systematic Reviews of Interventions [18]. Any disagreement between the two reviewers was resolved by a third reviewer for the assessment. The checklist by Philips et al. evaluates three domains of a model: (1) structure, (2) data, and (3) consistency.

## Data extraction and analysis

If a decision model was found to be associated with multiple studies, these studies were assessed as sharing the same parent model: Only the primary study (the study that described the model in greater detail) for each model was considered for the review, while supplementary and subsequent studies were documented as secondary studies. Data from secondary studies were not extracted. Data from the identified studies included in the review were extracted into data extraction grids (supplementary material Appendix 2) by two independent reviewers (L.J. and B.Y.). The extracted information included basic information, study details, population characteristics, basic modeling methodologies, model structure, data inputs for the included applications, model outcomes, model validation, and uncertainty.

## Results

**Fig. 1:**
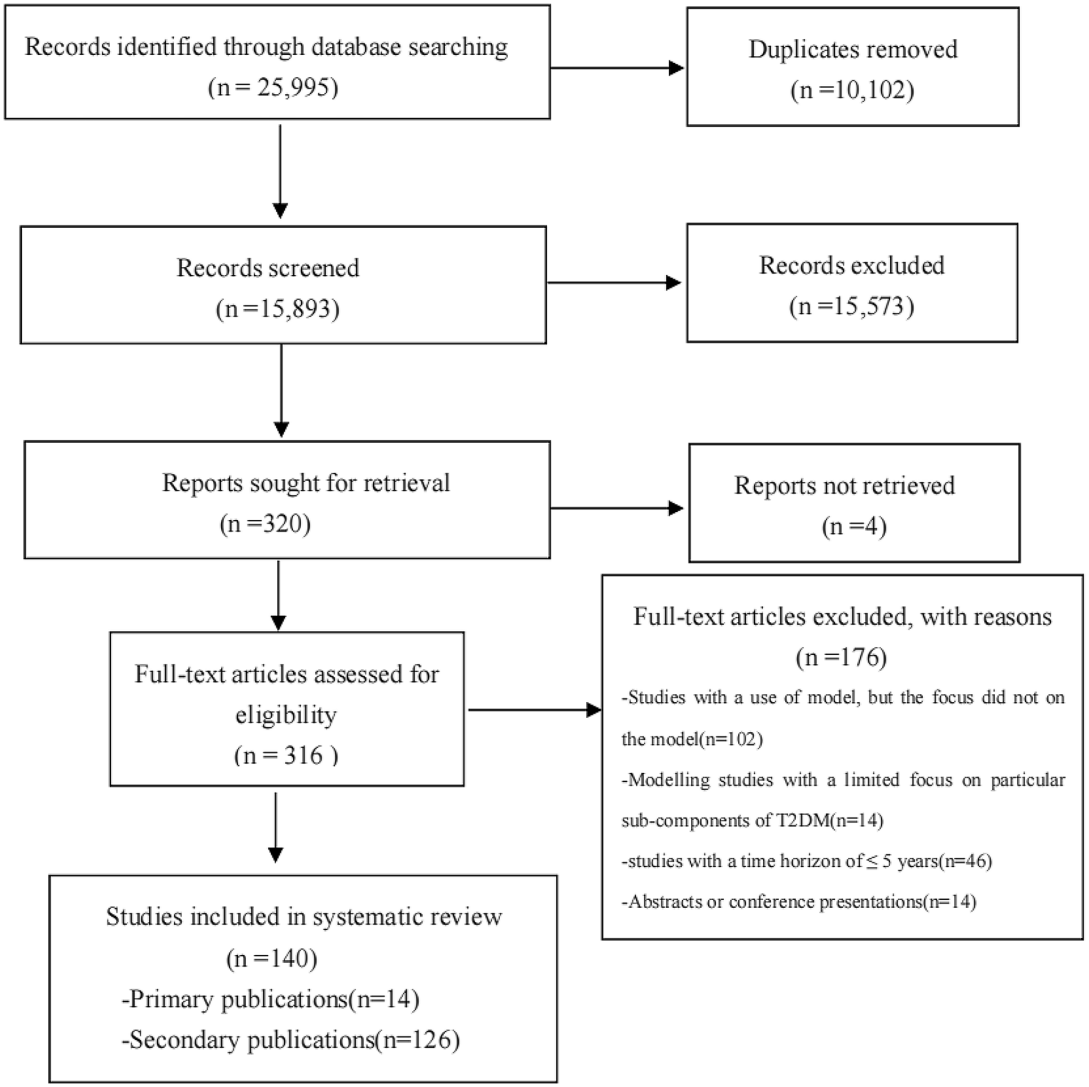
Flow diagram of literature search

A total of 25,995 related studies were searched in this systematic review; 10,102 duplicates were removed, and 15,893 studies were excluded based on first-pass screening using the title and abstract. Following the full-text review, 140 identified studies involving 14 decision models in T2DM were identified. Figure 1 shows the flow of studies throughout the review. Among the 140 identified studies, 79 used the CORE Diabetes Model (CDM), 17 used the Cardiff model, 13 used the United Kingdom Prospective Diabetes Study Outcomes Model 1 (UKPDS-OM1), 5 used the Archimedes model, 4 used the UKPDS-OM2, 4 used the Swedish Institute of Health Economics Cohort Model of Type 2 Diabetes (IHE), 3 used the Economic and Health Outcomes Model for T2DM (ECHO), 3 used the Michigan model, 3 used the Diabetes Cost-Effectiveness Model (DCEM), 2 used the Chinese Outcomes Model for T2DM (COMT), 2 used the Non-Insulin-Dependent Diabetes Mellitus model (NIDDM), 2 used the Sheffield model, 2 used the Ontario Diabetes Economic Model (ODEM), and 1 used the Cornerstone Diabetes Simulation model (CDS). For each model, only the primary studies that described the model in greater detail were considered for review, and supplementary and subsequent studies were documented as secondary studies. The list of secondary studies is summarized in supplementary material Appendix 3. Models were set in the USA (*n* = 3) [9, 19, 20], UK (*n* = 3) [10, 21, 22], Sweden (*n* = 2) [23, 24], Canada (*n* = 2) [11, 25], China (*n* = 1) [26], Switzerland (n = 1) [27], Australia (*n* = 1) [28], and in multiple countries (*n* = 1) [12]. Four models [9, 12, 20, 27] solely utilized Markov chains, seven models [^11, 19, 21, 22, 25, 26, 28^] solely utilized risk equations, and three models [10, 23, 24] utilized both of them. Except for the Archimedes model, all other models (*n* = 13) implemented an annual cycle length. The time horizon of most models is flexible, up to the course of a lifetime. Almost all models involved cost-utility or cost-effectiveness analysis. An overview of each model is outlined in Tables 1 and 2 sorted by year of publication.

**Table 1:** Overview of characteristic of decision models in type 2 diabetes (sorted by year of publication)

Model	Publication	Model perspective	Model design	Simulation	Cycle	Time horizon
Name	(year)	(base case)	(type of model)	method	length	
NIDDM [20]	1997	Patient	Markov	Patient level	Annual	Flexible (up to lifetime)
DCEM [12]	2002	Healthcare system	Markov	Cohort level	Annual	Lifetime or age 95
Archimedes [19]	2003	NR	Differential equations	Patient level	Continuous in time	Flexible (up to lifetime)
CDM [27]	2004	Healthcare payer	Markov	Cohort /patient level	Annual	Flexible (up to lifetime) (Exception: Foot ulcer sub model [1 month] model [3 months])
UKPDS-OM1 [21]	2004	Healthcare system	Differential risk model equations	Patient level	Annual # (Smoking status was based on 3-year periods from diagnosis of diabetes)	Lifetime
Michigan [9]	2005	Healthcare system	Markov	Patient level	Annual	Flexible (up to lifetime)
Cardiff [10]	2006	Healthcare system	Markov + Differential risk model equations	Patient level	Annual	Flexible(up to lifetime)
ODEM [11]	2007	Healthcare system (the Ontario Ministry of Health and Long-Term Care)	Differential risk model	Patient level	Annual	Flexible (up to lifetime)
Sheffield [22]	2010	NHS and personal social services	Differential risk model equations	Patient level	Annual	Lifetime
UKPDS-OM2 [28]	2013	Healthcare system	Differential risk model equations	Patient level	Annual	Lifetime
ECHO [24]	201	NR	Markov + Differential risk model equations	Patient level	Annual	Flexible (up to lifetime)
IHE [23]	2018	Healthcare decision-makers	Markov + Differential risk model equations	Cohort level	Annual	Flexible (maximum of 40 years)
COMT [26]	2018	Healthcare system	the latest risk Equations	Patient level	Annual (Exception: clinical neuropathy [1 month])	Lifetime
CDS [25]	2019	Healthcare decision-makers	Differential risk model equations	Patient level	Annual	Flexible (maximum of 100 years

**NIDDM** the Non-Insulin-Dependent Diabetes Mellitus model, **DCEM** the Diabetes Cost-Effectiveness Model, **CDM** the CORE Diabetes Model, **UKPDS-OM1/2** the United Kingdom Prospective Diabetes Study Outcomes Model 1/2, **ODEM** the Ontario Diabetes Economic Model, **ECHO** the Economic and Health Outcomes Model for T2DM, **IHE** the Swedish Institute of Health Economics Cohort Model of Type 2 Diabetes, **COMT** the Chinese Outcomes Model for T2DM, **CDS** the Cornerstone Diabetes Simulation model, **NR** not reported

**Table 2:** Overview of characteristic of decision models in type 2 diabetes (sorted by year of publication)

Model	Intervention and comparator	Basic data entered	Risk factors (base case)	Discounting	Model outcomes
Name	(base case)				
NIDDM [20]	NR	Age, sex, ethnicity, age at diagnosis of diabetes	Age, BMI, smoking, race, cholesterol, BP, income, physical activity, stress score marital status, occupation and family history of MI	NR	LY, ICER, costs, the cumulative incidence of complications
DCEM [12]	Intensive Glycemic control and conventional treatment	Age, sex, ethnicity, hypertension status, hypercholesterolemia status and current smoking status	NR	3% per-annual	LY, ICER, QALY, the number of discounted QALYS, costs the cumulative incidence of complications
Archimedes [19]	Three main types of treatments (1) Insulin; (2) Oral drugs; (3) Lifestyle (diet and exercise)	NR	NR	NR	LY, ICER, QALY, costs, the cumulative incidence of complications, expected
CDM [27]	Multiple interventions (1) Conventional therapy, (2) Intensive therapy	Age, sex, ethnicity, duration of diabetes, HbA1c, smoking, BP, BMI, Lipid levels, baseline complications	Age, BMI, HbA1c, SBP, T- CHOL, HDL, LDL, TRIG, smoking, alcohol consumption, duration of diabetes	NR	LY, ICER, QALY, costs, the cumulative incidence of complications, an accept- ability curve and/or NHB
UKPDS-OM1 [21]	(1) Conventional blood glucose control; (2) Intensive blood glucose control	Age, sex, ethnicity, HbA1c, BMI, smoking, BP, HDL age at diagnosis of diabetes, atrial fibrillation at diagnosis, PVD at diagnosis, history of diabetes related events, risk factors	HbA1c, SBP, HDL, smoking	NR	LY, QALY, costs, the cumulative incidence of complications
Michigan [9]	(1)diet and exercise; (2) oral anti-diabetic (3) insulin	Age, sex, ethnicity, HbA1c,BMI, smoking, SBP, age at diagnosis of diabetes, length of time in the current health, hypertension, serum total cholesterol level	NR	NR	Health utility scores, costs, the cumulative incidence of complications
Cardiff [10]	NR	Age, sex, ethnicity, smoking, duration of diabetes, risk factors	HbA1c,SBP,HDL, Weight, total cholesterol	6% per-annum (costs) 1.5% per-annum (benefits)	QALY, cost, total number of clinical events
ODEM [11]	A multidisciplinary primary care diabetes management program	Age, sex, ethnicity, HbA1c,BMI, smoking, SBP, DBP, HDL, total cholesterol, age at diagnosis diabetes, medical history, history of other medical conditions	HbA1c,SBP,HDL, total, cholesterol, smoking	3% per-annual	LY, ICER, QALY, costs, the cumulative incidence of complications
Sheffield [22]	DESMOND intervention	Age, sex, ethnicity, HbA1c, BMI, smoking, SBP, HDL, total cholesterol, age at diagnosis of diabetes, therapy at entry	HbA1c,BP, lipid concentration, smoking	3.5% per annum	LY, ICER, QALY, costs, CEAC, the cumulative incidence of complication
UKPDS-OM2 [28]	(1) Conventional blood glucose control; (2) Intensive blood glucose control;	Demographic factors(age, sex, BMI, ethnicity, duration of diabetes), risk factors, event history	HbA1c,SBP,HDL,LDL, eGFR, HR, PVD, smoking, WBC, atrial fibrillation, albuminuria, hemoglobin	NR	LY, QALY, costs, annual incidence of death or complications
ECHO [24]	Anti-diabetes treatment	Age, sex, HbA1c,BMI,SBP, HDL, duration of diabetes, history of pre-existing micro- and macro-vascular disease	Same with “basic data entered”	NR	LY, ICER, QALY, costs, mean survival, NMBs
IHE [136]	(1)Improved lifestyle patterns; (2)drug therapy	Age, sex, ethnicity, HbA1c,BMI, smoking, SBP, DBP, HDL, LDL, TC, WBC, HR, eGFR, duration of disease	Demographics(age, gender, ethnicity),biomarkers(HbA1c, SBP, DBP, TC, LDL, HDL, BMI, WBC, HR, eGFR), Pre-existing complications	NR	LY, ICER, QALY, NMBs the cumulative incidence of complications
COMT [147]	Anti-diabetic therapy	Age, sex, ethnicity, HbA1c, HDL, smoking, BP, history of cardiovascular disease, medication history, SR, urine albumin/creatinine ratio	Age, sex, ethnicity, smoking, BMI, SBP, total/HDL cholesterol age at diagnosis diabetes, history of diabetes complications	5% per-annual	LY, ICER, QALY, cost DALY, the cumulative incidence of complications
CDS [154]	NR	Age, sex, ethnicity,HbA1c,BMI, smoking, SBP, HDL, LDL,HR, hemoglobin, albuminuria, PVD, eGFR, WBC, the baseline complications, age at diagnosis diabetes	Age, sex, ethnicity, smoking, HbA1c,BMI,SBP,HR, LDL,HDL, hemoglobin, albuminuria, PVD, eGFR, WBC	NR	LY, ICER, QALY, cost, the cumulative incidence of complications

**BMI** Body Mass Index, **BP** blood pressure, **CEAC** cost-effectiveness acceptability curve, **DBP** diastolic blood pressure, **DALY** disability-adjusted life-year, **eGFR** estimated glomerular filtration rate, **HR** heart rate, **HDL** high-density lipoprotein, **ICER** incremental cost-effectiveness ratios, **LY** life year, **LDL** low-density lipoprotein cholesterol, **MI** myocardial infarction, **NHB** net health benefit, **NMB(s)** net monetary benefit(s), **PVD** peripheral vascular disease, **QALY** quality-adjusted life year, **SBP** systolic blood pressure, **T-CHOL/TC** total cholesterol, **TRIG** triglycerides, **WBC** white blood cell**, NR** not reported

### Model structure

Tables 1 and 2 show aspects of model structures. Eight model structures [10–12, 22, 23, 25, 26, 28] were constructed in reference to pre-existing models. Models had certain differences in how health states were divided (Tables 3 and 4). The DCEM model placed greater emphasis on macrovascular complications, whereas the NIDDM and Michigan models placed greater emphasis on microvascular complications. Other models, apart from the Archimedes model, emphasized both macrovascular and microvascular complications (CDM, UKPDS OM1/2, IHE, ODEM, Cardiff, Sheffield, CDS, COMT, ECHO). The Archimedes model has no clear-cut health states, as it is continuous in time, with no discrete time steps, and any event could occur at any time. The IHE model included numerous health states for complications and used two parallel Markov chains. The first chain consisted of 120 different microvascular health states, and the second chain was made up of 100 different macrovascular health states. Six models [19, 22–24, 26, 27] included adverse events. Almost all these models classified them as treatment outcomes, not as independent health states. However, the CDM model incorporated adverse events into the model as independent health states. All models included death as a health state, while each model had different levels of detail in this state.

**Table 3:** Summary of model health states and adverse events

Model	CHD	Nephropathy	Retinopathy	Neuropathy
Name				
NIDDM [20]	CVD (No CVD,CVD morbidity and mortality)	No nephropathy, MA 0.03–0.3 g/l (American Indians 30–299 mg/g Creatinine), proteinuria > 0.4 g/1 ESRD	No retinopathy, non-proliferative retinopathy, PDR, significant ME, visual acuity < 20/100 in better eye	No neuropathy, symptomatic neuropathy, first LEA
DCEM [12]	Normal, CHD, angina, history of CA/MI, CA/MI, death	Normal, low micro/high micro, clinical nephropathy, ESRD, ESDR death	Normal, photocoagulation, blind	Normal, peripheral neuropathy LEA, history of LEA, subsequent LEA, LEA death
Archimedes [19]	NA	NA	NA	NA
CDM [27]	MI (no history of MI, history of MI, death following MI), angina (no angina, history of angina), CHF (no CHF, history of CHF, death following CHF)	No renal complications, microalbuminuria, gross proteinuria, ESRD, death following ESRD	No retinopathy, BDR, PDR SVL, Macular edema (no macular edema, macular edema), cataract (no cataracts, first cataract with operation, second cataract with operation)	No neuropathy, neuropathy PVD(no PVD, PVD)
UKPDS- OM1 [21]	MI (non-fatal MI, fatal vascular cardiac event, sudden death), IHD, CHF	Creatinine levels of above 250 Snellen, 6/60 ETDRS log MAR 1.0, any acute inter-current illness, death due to renal failure	Blindness in one eye (a visual acuity of a digit or limb, fatal worse for any reason < persisting for > 3 months)	Amputation (first amputation# peripheral vascular event)
Michigan [9]	Normal, angina, MI/cardiac arrest, history of MI/cardiac arrest, death due to CVD	Normal, microalbuminuria, proteinuria, ESRD with dialysis ESRD with transplant, death due to ESRD	Normal, non-proliferative retinopathy, proliferative retinopathy, macular edema blindness	Normal, clinical neuropathy, amputation
Cardiff [10]	MI (non-fatal MI, fatal MI)	ESRD, MA, GPR subsequent years SVL/blindness	First year SVL/blindness, PVD (without amputation, with amputation)	Symptomatic neuropathy, LEA,
ODEM [11]	IHD (non-fatal IHD, fatal IHD), MI (non-fatal MI, fatal MI),heart failure (non-fatal, fatal)	Renal failure (fatal renal failure, non-fatal renal failure)	Blindness (non-fatal, fatal)	Amputation (non-fatal, fatal)
Sheffield [22]	CHD, heart failure	NR	NR	NR
UKPDS- OM2 [28]	MI (non-fatal MI, fatal MI, sudden death), IHD, CHF, second-event for MI,IHD,CHF	Same with the UKPDS-OM1 model nephropathy health state	Same with the UKPDS-OM1 model retinopathy health state	Same with the UKPDS-OM1 model neuropathy health state + second events for amputation
ECHO [24]	IHE, MI, CHF	No nephropathy, MA, GPR, ESRD	No retinopathy, BDR, PDR PDR & blind, ME, ME & PDR, ME & blind, ME & PDR & blindness, in 1 eye, blindness in both eyes	No neuropathy, symptomatic, PVD, symptomatic /PVD, foot ulcer, LEA, subsequent LEA
IHE [23]	MI (none, first MI, post-first MI, subsequent Mis, post subsequent MIs),IHD (None, IHD), CHF (None, CHF)	None, Microalbuminuria, Macroalbuminuria, ESRD	None, BDR, PDR, ME, ME and PDR, SVL	None, PVD, LEA, Post LEA
COMT [26]	MI, CHF, ASCVD, CVD, CVD death	ESRD	Blindness	Clinical neuropathy, amputation (minor, major)
CDS [25]	CHF, IHD, MI	Renal failure	Blindness	Amputation

**ASCVD** arteriosclerotic cardiovascular disease, **BDR** background diabetic retinopathy, **CA** cardiac arrest, **CHD** coronary heart disease, **CHF** congestive heart failure, **CVD** cardiovascular disease, **ESRD** end-stage renal disease, **GPR** gross proteinuria, **IHD** ischemic heart disease, **LEA** lower extremity amputation, **MA** microalbuminuria, **ME** macular edema, **MI** myocardial infarction, **PDR** proliferative retinopathy, **PVD** peripheral vascular disease, **SVL** severe visual loss, **NA** not applicable, **NR** not reported

**Table 4:** Summary of model health states and adverse events

Model	Stroke	Foot ulcer	Others	Adverse events
Name				
NIDDM [20]	NR	NR	Mortality (CVD mortality, Non- CVD mortality)	NR
DCEM [12]	Normal, stroke, history of Stroke, death		Death (die from LEA, ESRD, CHD, stroke, or from other causes unrelated to diabetes)	
Archimedes [19]	NR	NR	The Archimedes model is a person-by-person, object-by-object simulation written in hundreds of differential equations that mathematically represent physiological pathways and the effects of multiple diseases, tests and treatments. No clear-cut health- states available	Hypoglycemia
CDM [27]	No history of stroke, history	No foot ulcer, uninfected ulcer infected ulcer, healed ulcer uninfected recurrent ulcer, infected recurrent ulcer, gangrene history of amputation	Non-specific mortality (alive and death)	Hypoglycemia (alive with hypoglycemia, death from hypoglycemia), lactic acidosis (alive with lactic acidosis, death from lactic acidosis) from lactic acidosis)
UKPDS- OM1 [21]	First non-fatal stroke, fatal stroke	N	Death (death in the first year with complications, death from causes unrelated to diabetes)	N
Michigan [9]	Normal, stroke, history of stroke death due to stroke	NR	Mortality (die from ESRD, stroke CHD, non-renal &non-cardiovascular)	NR
Cardiff [10]	First non-fatal stroke, fatal stroke	NR	Death	NR
ODEM [11]	Fatal Stroke, non-fatal stroke	NR	Death	NR
Sheffield [22]	Stroke (status not specified)	NR	Death (diabetes and other cause mortality)	Weight gain edema & reversible heart failure, hypos
UKPDS- OM2 [28]	First non-fatal stroke, fatal stroke second events for stroke	Diabetic ulcer (Ulcer of the lower limb)	Same with the UKPDS-O1 model ‘others’ health state	NR
ECHO [24]	Stroke	Categorize it into neuropathy	Mortality (event fatality, diabetes mortality, other mortality)	Hypos (moderate, severe), other AEs ((peripheral edema, Osteoporosis, Urinary tract disorders, vaginitis)
IHE [23]	None, first stroke, post first stroke, subsequent strokes, post subsequent strokes	NR	Mortality (event mortality, diabetes mortality and other mortality)	Hypoglycemia (mild, moderate and severe), three user-specified grades of hypoglycemia and five other user-specified adverse events
COMT [26]	Stroke	Uncomplicated DFU, complicated DFU	Death	Hypoglycemia
CDS [25]	Stroke	Foot ulcer	Mortality	NR

**CHD** coronary heart disease, **CVD** cardiovascular disease, **DFU** Diabetic foot ulcer, **ESRD** end-stage renal disease, **LEA** lower extremity amputation, **NR** not reported

Eleven identified models were patient-level simulation models, while cohorts were used in the DCEM and IHE models. Either the patient -or cohort-level simulation method can be used in the CDM model. Except for the Archimedes model and the ECHO model, others illustrated the model perspective in the primary citations. Ten models considered a healthcare-related perspective in the base case (7 models [9–12, 21, 26, 28] used a healthcare-system perspective, 2 models [23, 25] used a healthcare decision-maker perspective, and 1 model [27] used a healthcare-payer perspective), while the NIDDM and Sheffield models considered a patient perspective and a social perspective, respectively.

Thirteen models used an annual cycle length, while the Archimedes model was continuous in time. Three models [21, 26, 27] did not use an annual cycle length for specific health states. The time horizon of 9 models [9–11, 19, 20, 23–25, 27] was defined by users, up to one’s lifetime, while the time horizon of 5 models [12, 21, 22, 26, 28] was set to one’s lifetime. The transition probabilities between models varied in complexity. Risk equations were applied in most models to handle transition probabilities depending on the epidemiology of T2DM, the risk factors, the incidence and prevalence of diabetic complications, and comorbidities.

### Incorporation of risk factors

Eleven models [10, 11, 20–28] simulated annual changes in risk factors such as body mass index (BMI), glycemia, HbA1c, blood pressure (systolic and/or diastolic), and lipids (total cholesterol and/or high-density lipoprotein) (Table 2). The simulated trajectory of risk factors could affect the subsequent occurrence or development of diabetes and its complications. The DCEM and COMT models precisely controlled risk factors to reduce the onset and development of diabetes and its complications.

### Model outcomes

The major model outcomes are summarized as follows (Table 5):

**Table 5:** Summary of model outcomes

Model	LYs	ICER	QALYs	Costs		NMBs	Others
Name				Direct costs	Indirect costs		
NIDDM [20]	√	√	√	√			
DCEM [12]	√	√	√	√			The number of discounted QALYs
Archimedes [19]	√	√	√	√			Expected number of cases
CDM [27]	√	√	√	√	√		Acceptability curve and/or NHBs
UKPDS-OM1 [21]	√		√	√ (not classified direct or indirect)			
Michigan [9]				√			Health utility scores
Cardiff [10]			√	√ (not classified direct or indirect)			Total number of clinical events
ODEM [11]	√	√	√	√			
Sheffield [22]	√	√	√	√			CEAC
UKPDS-OM2 [28]	√		√	√ (not classified direct or indirect)			
ECHO [24]	√	√	√	√		√	Mean survival
IHE [23]	√	√	√	√	√	√	
COMT [26]	√	√	√	√			DALYs
COMT [26]	√	√	√	√			

**LYs** life years**, ICER** incremental cost-effectiveness ratios**, QALYs** quality-adjusted life years**, NMBs** net monetary benefits, **CEAC** cost-effectiveness acceptability curve, **DALYs** disability-adjusted life

years

Twelve models [11, 12, 19–28] reported life-years (LYs), ten model [11, 12, 19, 20, 22–27] reported incremental cost-effectiveness ratios (ICERs), and thirteen models [10–12, 19–28] reported quality-adjusted life years (QALYs). The ECHO and IHE models also reported net monetary benefits (NMBs). Some models [9, 10, 12, 19, 22, 24, 26, 27] also reported other outcomes.

### Cost

All models reported costs, albeit at different levels of detail. Eleven models [9, 11, 12, 19, 20, 22–27] reported direct costs, whereas the CDM and IHE models reported both direct and indirect costs. Three models (UKPDS OM1/2 and the Michigan model) did not describe cost in detail. The outcomes of three models (UKPDS OM1/2 model and the Michigan model) included costs, but none of the included studies classified costs into direct and indirect costs.

### Health utility

All models reported utility values as outcomes. Thus, subsequent cost-utility analyses (CUA) could be performed. Each health state in a model had a corresponding utility value. Utility values for complications were obtained with the EQ-5D health status questionnaire [10, 21, 28] and the Quality of Well Being–Self-Administered questionnaire (QWB-SA) [9]. Most CUA were made by calculating QALYs. Some models [11, 12, 19, 20, 22–27] also took ICERs into account and thus could perform incremental analyses.

### Main data sources for complications

All models reported some main data sources used to develop the health states of complications. The data commonly used to develop macrovascular complications included the Framingham datasets [20, 27] and the UKPDS [9, 10, 12, 19, 21–23, 27, 28]. For microvascular complications, the data sources were more complicated, and the commonly used sources were the Wisconsin Epidemiological Study of Diabetic Retinopathy (WESDR) [20, 27] and the UKPDS [27]. More than half of the models applied multiple data sources for each complication, while the remaining models only contained one or two data resources (Table 6).

**Table 6:** Summary of main data sources for diabetic complications

Model	CHD	Nephropathy	Retinopathy	Neuropathy	Stroke	Others
Name						
NIDDM [20]	The Framingham (CVD) [33]	WESDR [34], the Rochester Epidemiology Project [35]	WESDR [36, 37]	NHANES II [38], the Rochester Study (LEA) [39]	NR	NR
DCEM [12]	Weinstein MC et al. [40], Anderson KM et al. [41], Hunink MGM et al. [42]	NR		NR	NR	Mostly from UKPDS [21], Eastman et al. [40, 43]
Archimedes [19]						‘Features’ derived
CDM [27]	CVD: the Framingham [44] UKPDS [21], Herlitz et al. [45], the DIGAMI study [46]) Angina: the Framingham [166] CHF: the Framingham [44] PVD: the Framingham [44], PVD: the Framingham [44],	Wolfe RA et al. [47]	WESDR [36, 48], EURODIAB study [49] Cataract: UKPDS [50]	Partenen et al. [51, 52]	Petty et al. [53] Sprafka et al. [54]	Foot ulcer: Tennvall and Apelqvist [55] Hypoglycaemia: Poland and Israel 56, 57
UKPDS- OM1 [21]						All from UKPDS [21]
Michigan [9]	CHD:UKPDS [58], et al. [59], Ulvenstam G et al. [60], Lowel H et al. [61]Stevens RJ et al. [62]	Malmberg K Gall MA et al. [63] Ballard DJ et al. [35], Ravid M M et al. [64]	Klein R et al. [37, 65, 66], Moss SE et al. [67, 68]	Sands ML et al. [69], Adler AI et al. [70]	UKPDS [58], Hier DB et al. [71], Sacco RL et al. [72], Kothari V et al. [73]	Mortality: UKPDS [58]
Cardiff [10]				Cardiff data [74]		Mostly from UKPDS [75]
ODEM [11]						All from GHC
Sheffield [22]	UKPDS [62]	DCCT [76]	NR	NR	UKPDS [73]	NR
UKPDS- OM2 [28]						All from UKPDS [21, 28]
ECHO [24]	UKPDS [21]	Eastman et al. [43]	Eastman et al. [43]	Eastman et al. [43], Bagust et al. [77]	UKPDS [21]	NR
IHE [23]	Macrovascular: NDR [78], UKPDS [21, 28]	Bagust A et al. [77]	Bagust A et al. [79]	Eastman R.C.et al. [20]	NR	Mortality: UKPDS [21, 28]
COMT [26]	Gerstein HC et al.^80^ Wing RR et al. [81]	NR	NR	NR	NR	Perreault L et al. [82]
CDS [25]						Mostly from ADVANCE [83] LDS [84], THIN^85^

**NR** not reported

### Model validation

Eleven of fourteen primary studies reported that one or more validation checks had been performed. Four studies [10, 24, 26, 28] presented model face validation, eleven studies [9, 10, 19–21, 23–28] presented internal validation, ten studies [10, 19–21, 23–28] presented external validation, while cross-validation was conducted by three studies [24, 25, 28]. However, none of the 14 studies demonstrated predictive validation. Primary studies using the DCEM, ODEM, and Sheffield models did not report aspects of model validation (Table 7).

**Table 7:** Summary of model validation (data only extracted from 14 primary citations: for baseline cases)

Model	Face validation	Internal validation	External validation	Cross-validation	Predictive validation
Name					
NIDDM [20]		√	√		
DCEM [12]	NR	NR	NR	NR	NR
Archimedes [19]		√	√		
CDM [27]		√	√		
Michigan [9]		√			
Cardiff [10]	√	√	√		
ODEM [11]	NR	NR	NR	NR	NR
Sheffield [151]	NR	NR	NR	NR	NR
UKPDS-OM2 [28]	√	√	√	√	
ECHO [24]	√	√	√	√	
IHE [23]		√	√		
COMT [26]	√	√	√	√	
CDS [25]		√	√	√	

**NR** not reported (for baseline cases)

### Model uncertainty

Eleven models [9–12, 20–23, 25, 27, 28] were able to deal with model uncertainty, which was described in varying levels of detail in the primary studies. One-way sensitivity analysis was run in the Cardiff, DCEM, ODEM, and UKPDS-OM2 models. Based on 14 primary studies, none of the models reported a multi-way sensitivity analysis. Probabilistic sensitivity analysis (PSA) capabilities were reported by 9 models (NIDDM, DCEM, CDM, UKPDS-OM1/2, Michigan, Sheffield, IHE, COMT). Five models [9, 20, 25, 27, 28] used the Monte Carlo technique for PSA, while three models [12, 21, 27] used the nonparametric bootstrap method. Only 3 model [23, 27, 28] clearly indicated whether first-order or second-order uncertainty was performed (Table 8).

**Table 8:** Summary of model uncertainty (data only extracted from 14 primary citations: for baseline cases)

Model	One-way sensitivity analysis	Multi-way sensitivity analysis	probabilistic sensitivity analysis
Name			(PSA)
NIDDM [20]			√ Use Monte Carlo simulations
CDC-RTI [12]			√ The nonparametric bootstrap method is used
Archimedes [19]	NR	NR	NR
CDM [27]			√ The nonparametric bootstrap method is used + first and second-order Monte Carlo simulations
UKPDS-OM1 [21]			√A combination of bootstrap methods and multiple imputation methods were used √ Use Monte Carlo simulations
Michigan [9]			√ Use Monte Carlo simulations
Cardiff [10]	√		
ODEM [11]	√		
Sheffield [22]			√
UKPDS-OM2 [28]	√		√ use Monte Carlo or first order uncertainty + Parameter or second order uncertainty
ECHO [24]	NR	NR	NR
IHE [23]			√ Second order PSA
COMT [26]	NR	NR	NR
CDS [25]			√ use Monte Carlo simulations

**NR** not reported (for baseline cases)

### Model quality

**Fig. 2:**
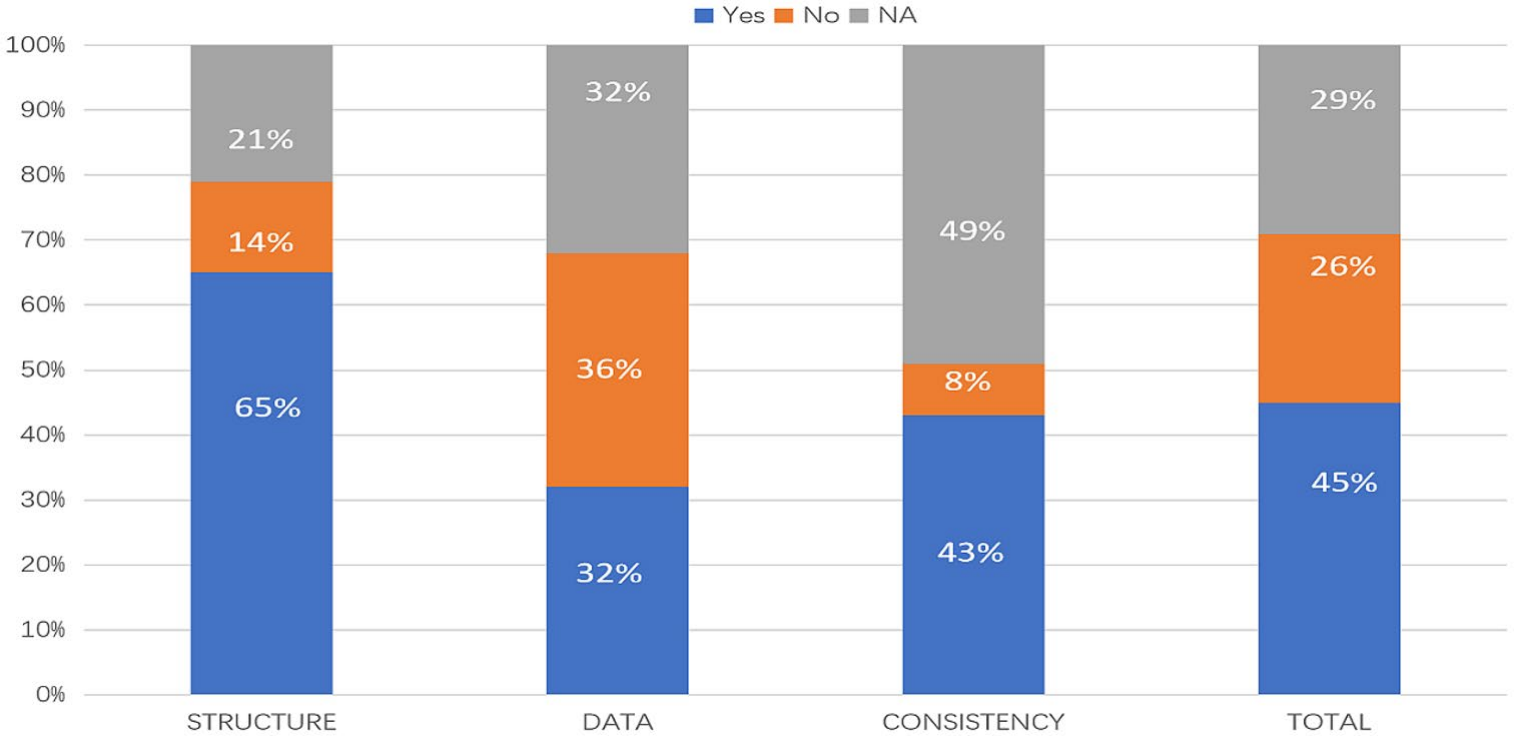
Quality of modeling studies according to the Phillips checklist. Legend: A “yes” answer was assigned if a criterion was fulfilled. A “No” answer was assigned to criteria that were not fulfilled. NA indicates not applicable

In accordance with the checklist from Philips et al. [17], the percentage of fulfilled criteria was unequally distributed across studies and dimensions of quality (model structure, data, and consistency). Overall, 45% of the criteria were met, 26% were not met, and 29% were not applicable in the 14 primary studies. Figure 2 shows that on average across all included studies, model structure ranked the highest, with 65% of criteria for quality being met, followed by model consistency (43%) and model data (32%) (Tables 9, 10, and 11).

**Table 9:**
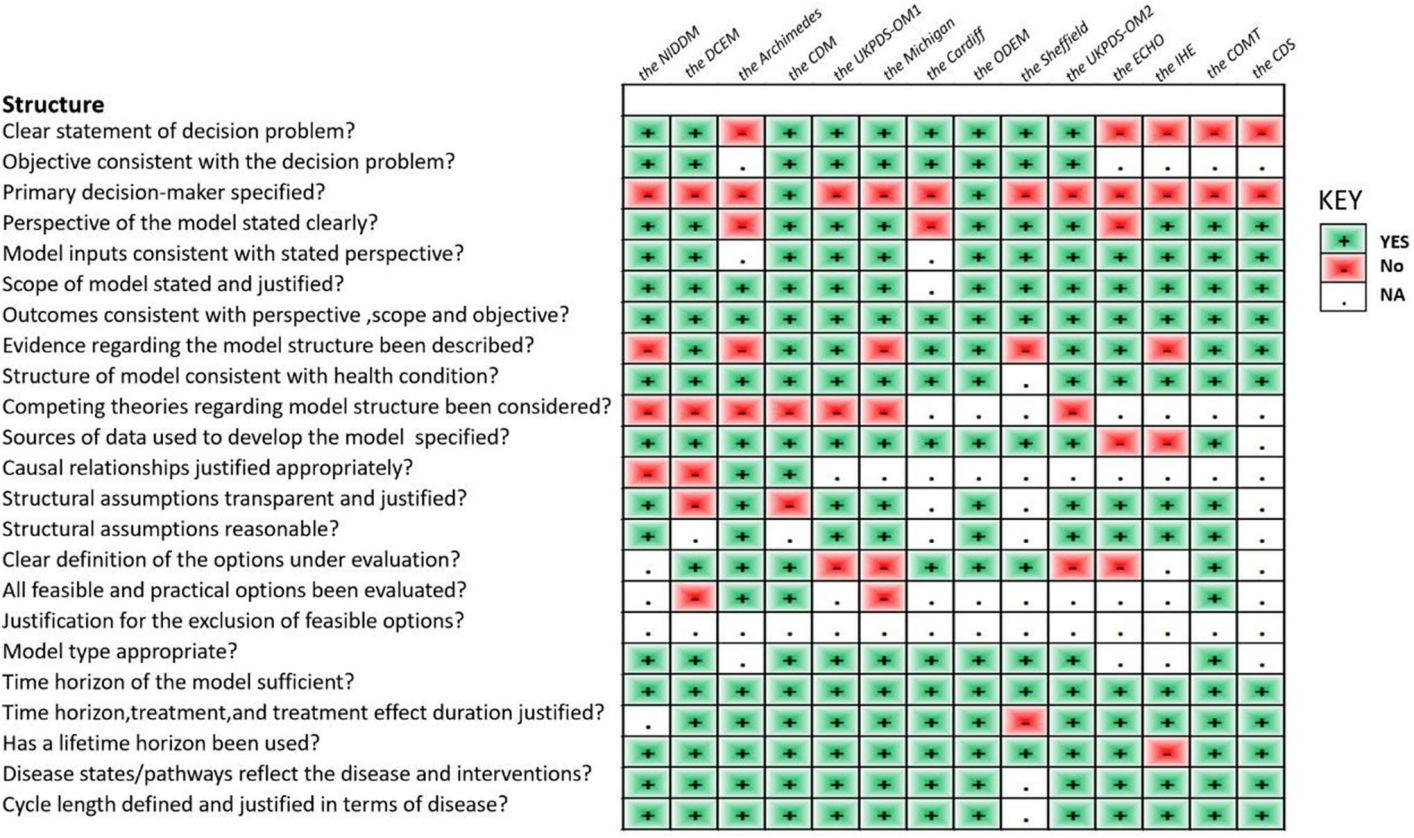
Philips checklist results

**Table 10:**
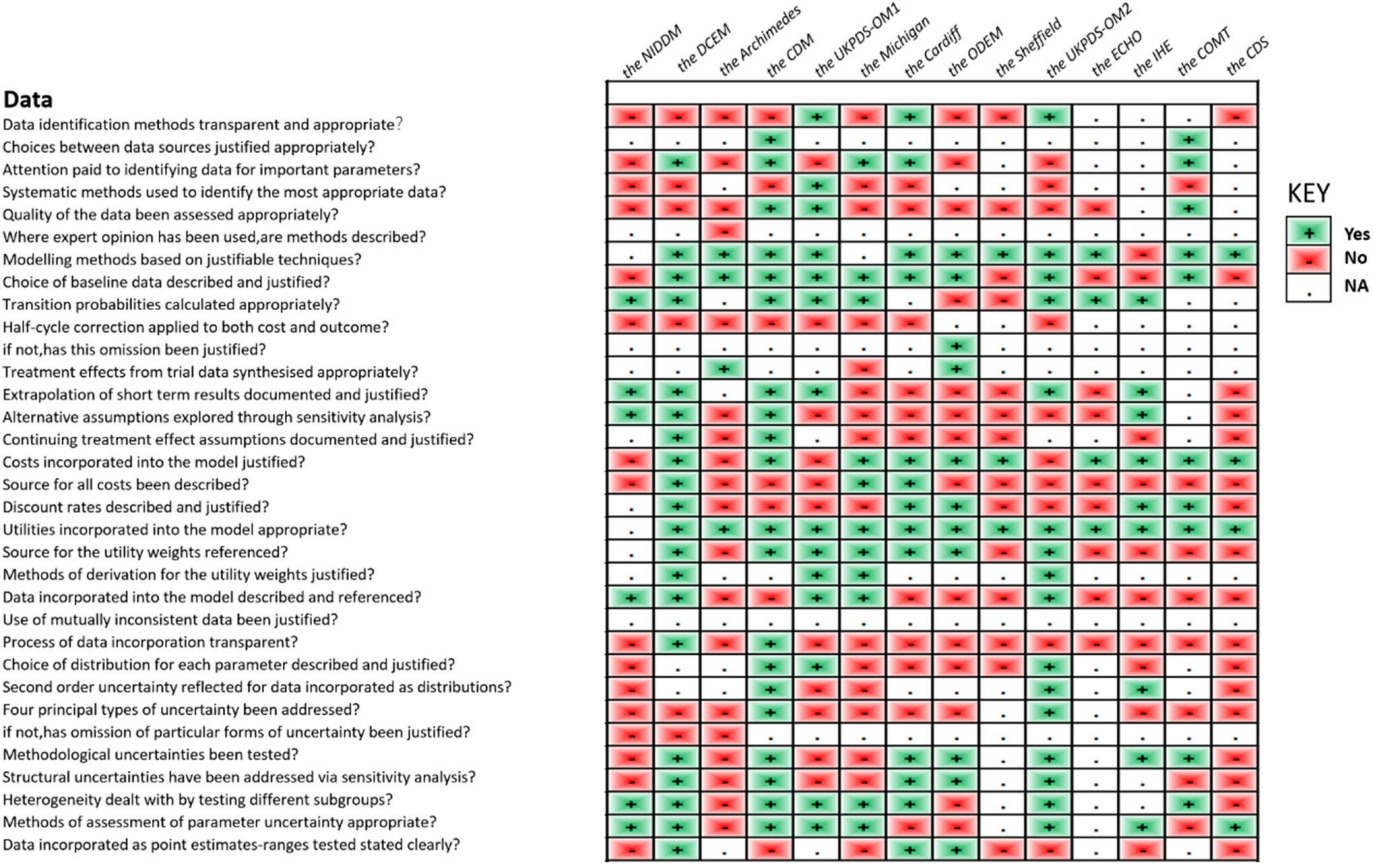
Philips checklist results

**Table 11:**
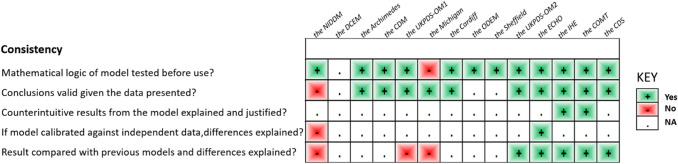
Philips checklist results

## Discussion

Our systematic review included 140 studies describing 14 decision models in T2DM. We extracted data from the primary studies for each model, and the remaining 126 studies were identified as secondary studies (Supplementary material Appendix 2). We found that there were fairly mature modeling technologies and relatively fixed model structures for existing decision models for T2DM. Overall, the 13 identified models (except for the Archimedes model) divided the disease into discrete health states, followed by establishing Markov chains or risk equations to simulate the lifelong course of the disease. However, the review of these studies showed that the existing T2DM models still had certain limitations in terms of quality and extrapolation.

Previous systematic reviews of T2DM models [29–32] have focused more on model outputs than on their capabilities. However, the primary focus of this systematic review was the capabilities of these models. Based on the characteristics of each model, we briefly summarized the more suitable models for different study demands as follows:

1. If a study focused on simulating the trajectory of T2DM and/or diabetic macrovascular complications (e.g., cardiovascular disease, angina, myocardial infarction, or cardiac arrest), the best choice is the DCEM model.
2. If the study focused on simulating the trajectory of T2DM and/or diabetic microvascular complications (e.g., retinopathy and/or nephropathy), the best choices are the NIDDM model or the Michigan model. It is worth noting that the NIDDM model was the first diabetes model and it is rarely used now, but it is still of great value in the development of diabetes models. Many current models were constructed based on the NIDDM model.
3. If the objective is to conduct a comprehensive study of the trajectory of T2DM and its various complications, the best choices are the CDM model, the UKPDS OM1/2 model, the IHE model, the ODEM model, the Cardiff model, the Sheffield model, CDS model, COMT model, or the ECHO model.
4. If the objective is to simulate a continuous trajectory of diabetes and its complications, the Archimedes model is the best choice.
5. If the study is aimed at Chinese and Asian populations, it is recommended to use the COMT model.
6. If the study focuses on risk factors, the UKDPS-OM1 or UKDPS-OM2 models can be considered for simulation.
7. To evaluate T2DM interventions where hundreds of simulations are routinely required (e.g., given multiple indications and treatment comparators and the need for extensive sensitivity analysis), the IHE model can be considered first, because the run times for the IHE model were short when compared to most T2DM microsimulation models.

In this systematic review, the 14 identified models were rather heterogeneous in terms of model structures, the main data sources used by models, and model uncertainty.

We observed that most model structures were composed of discrete health states, and each discrete state was simulated annually through transition probabilities. However, the Archimedes model applied a comprehensive approach to model structure by simulating the disease at the organ level; it has no clear-cut health states. The level of detail in the classification of health states was different between models, and not all models had a clear definition of each health state it contained. However, the desired level of complexity must be balanced with the required transparency. Despite variations in model structure and scope, there should be a reasonably clear consensus of what broad categories of health states should be considered in the same type of T2DM models.

Many of the data sources used in model development are older data sets, such as the UKPDS and Framingham datasets; this limitation also exists in T1DM models. Although this limitation is well known, these data sources are currently recognized as the best available sources for modeling. This review also found that most of the data inputted to models were based on European populations; only 1 of the 14 models was developed based on Asian population data (the COMT model). However, in the era of real-world evidence, with an increasing availability of registry data from clinical practice settings, model validation incorporating modern T2DM epidemiological data into disease progression equations for simulation will be important. The development of this technology may resolve the impacts of limitations on model simulation.

The level of description of model uncertainty varied among the included studies, and there is a lack of standardized terminology regarding model uncertainty in these studies. This may hinder the understanding of what has actually been carried out. For example, in studies conducting Monte Carlo simulation or PSA, it was not always clear whether the report considered first- or second-order uncertainty. This should be noted because many health technology assessment (HTA) agencies demand that second-order uncertainty be captured in PSA. However, it does require multiple and complex computer calculations to solve second-order uncertainty through the PSA of the microsimulation models. This may be why some studies have not clearly stated their uncertainty.

Although a rigorous systematic review was undertaken to identify all relevant studies of decision models in T2DM, some limitations of this review should be acknowledged. First, the data were extracted mainly through the primary study for each model, rather than the latest study, which may cause some of the latest views on models to be ignored. In general, ICERs were also obtained when calculating QALYs to perform CUA. However, in model outcomes, 13 models reported QALYs, and only 10 of these models reported ICERs. This may be due to the lack of data from secondary studies. A similar review should be conducted on secondary studies of each model to provide a more comprehensive evaluation of the included models. Second, models with a limited focus on particular sub-components of T2DM were excluded. Models focused on particular sub-components of T2DM may provide a more meticulous and complex simulation method. However, these models only involved specific components of T2DM, which may lead to failure to consider the connection of the various components of diabetes in modeling. Finally, the assessment of study quality may be biased, as some studies were not described in full detail because of word limits for publications.

## Conclusion

We conducted a comprehensive systematic review focusing on capabilities of the existing decision models for T2DM, and briefly summarized the more suitable models for different study demands. It is necessary to use decision models to simulate the lifelong course of diseases, especially for chronic diseases, to evaluate whether new technologies or interventions have values. A general conclusion from the review is that the existing decision models for T2DM were rather heterogeneous on the level of detail in the classification of health states. Thus, more attention should be focused on balancing the desired level of complexity against the required level of transparency in the development of T2DM decision models. Furthermore, we should consider including secondary studies for a more comprehensive systematic review.

### Registration

This systematic review was registered in the PROSPERO database (CRD42020171838).https://www.crd.york.ac.uk/prospero/display_record.php?ID=CRD42020171838

## Supplementary Information

Below is the link to the electronic supplementary material.

Supplementary file1 (DOCX 170 kb)

## Data Availability

Evaluated studies are publicly available peer-reviewed scientific publications.

## References

[1] International Diabetes Federation (2019) IDF Diabetes Atlas. 9th ed. Brussels: International Diabetes Federation; 2019. Available at http://www.diabetesatlas.org. Accessed Feb 2020

[2] Global, regional, and national disability-adjusted life-years (DALYs) for 333 diseases and injuries and healthy life expectancy (HALE) for 195 countries and territories, 1990–2016: a systematic analysis for the Global Burden of Disease Study 2016 (2017). Lancet 390(10100):1260–1344. 10.1016/s0140-6736(17)32130-x 10.1016/S0140-6736(17)32130-X PMC5605707 28919118

[3] Wang L, Gao P, Zhang M (2017). Prevalence and ethnic pattern of diabetes and prediabetes in China in 2013. JAMA.

[4] Chan JC, Zhang Y, Ning G (2014). Diabetes in China: a societal solution for a personal challenge. Lancet Diabetes Endocrinol.

[5] Xu Y, Wang L, He J (2013). Prevalence and control of diabetes in Chinese adults. JAMA.

[6] Dakin HA, Devlin NJ, Odeyemi IA (2006). "Yes", "No" or "Yes, but"? Multinomial modelling of NICE decision-making. Health Policy.

[7] Caro JJ, Briggs AH, Siebert U, Kuntz KM (2012). Modeling good research practices–overview: a report of the ISPOR-SMDM Modeling Good Research Practices Task Force–1. Value Health.

[8] Weinstein MC, O'Brien B, Hornberger J (2003). Principles of good practice for decision analytic modeling in health-care evaluation: report of the ISPOR Task Force on Good Research Practices-Modeling Studies. Value Health.

[9] Zhou H, Isaman DJM, Messinger S (2005). A computer simulation model of diabetes progression, quality of life, and cost. Diabetes Care.

[10] McEwan P, Peters JR, Bergenheim K, Currie CJ (2006). Evaluation of the costs and outcomes from changes in risk factors in type 2 diabetes using the Cardiff stochastic simulation cost-utility model (DiabForecaster). Curr Med Res Opin.

[11] O'Reilly D, Hopkins R, Blackhouse G (2007). Long-term cost-utility analysis of a multidisciplinary primary care diabetes management program in Ontario. Can J Diabetes.

[12] Hoerger TJ, Bethke AD, Richter A (2002). Cost-effectiveness of intensive glycemic control, intensified hypertension control, and serum cholesterol level reduction for type 2 diabetes. J Am Med Assoc.

[13] Kahn R (2004). Guidelines for computer modeling of diabetes and its complications. Diabetes Care.

[14] Leal J, Morrow LM, Khurshid W, Pagano E, Feenstra T (2019). Decision models of prediabetes populations: a systematic review. Diabetes Obes Metab.

[15] Henriksson M, Jindal R, Sternhufvud C (2016). A systematic review of cost-effectiveness models in type 1 diabetes mellitus. Pharmacoeconomics.

[16] Page MJ, McKenzie JE, Bossuyt PM (2021). The PRISMA 2020 statement: An updated guideline for reporting systematic reviews. PLoS Med.

[17] Philips Z, Bojke L, Sculpher M, Claxton K, Golder S (2006). Good practice guidelines for decision-analytic modelling in health technology assessment: a review and consolidation of quality assessment. Pharmacoeconomics.

[18] Wright D, Little R, Turner D, Thornley T (2019). Diabetes screening through community pharmacies in England: A cost-effectiveness study. Pharm (Basel).

[19] Eddy DM, Schlessinger L (2003). Archimedes: a trial-validated model of diabetes. Diabetes Care.

[20] Eastman RC, Javitt JC, Herman WH (1997). Model of complications of NIDDM I Model construction and assumptions. Diabetes Care.

[21] Clarke PM, Gray AM, Briggs A (2004). A model to estimate the lifetime health outcomes of patients with type 2 diabetes: the United Kingdom prospective diabetes study (UKPDS) outcomes model (UKPDS No. 68). Diabetologia.

[22] Gillett M, Dallosso HM, Dixon S (2010). Delivering the diabetes education and self management for ongoing and newly diagnosed (DESMOND) programme for people with newly diagnosed type 2 diabetes: Cost effectiveness analysis. BMJ.

[23] Lundqvist A, Carlsson KS, Johansen P, Andersson E, Willis M (2014). Validation of the IHE cohort model of type 2 diabetes and the impact of choice of macrovascular risk equations. PLoS ONE.

[24] Willis M, Asseburg C, He J (2013). Validation of economic and health outcomes simulation model of type 2 diabetes mellitus (ECHO-T2DM). J Med Econ.

[25] Su ZT, Bartelt-Hofer J, Brown S (2019). The use of computer simulation modeling to estimate complications in patients with type 2 diabetes mellitus: comparative validation of the cornerstone diabetes simulation model. Pharmacoecon Open.

[26] Wu B, Ma J, Zhang S, Zhou L, Wu H (2018). Development and validation of a health policy model of type 2 diabetes in Chinese setting. J Comparative Effect Res.

[27] Palmer AJ, Roze S, Valentine WJ (2004). The CORE Diabetes model: Projecting long-term clinical outcomes, costs and cost-effectiveness of interventions in diabetes mellitus (types 1 and 2) to support clinical and reimbursement decision-making. Curr Med Res Opin.

[28] Hayes AJ, Leal J, Gray AM, Holman RR, Clarke PM (2013). UKPDS Outcomes Model 2: a new version of a model to simulate lifetime health outcomes of patients with type 2 diabetes mellitus using data from the 30 year United Kingdom prospective diabetes study: UKPDS 82. Diabetologia.

[29] Asche CV, Hippler SE, Eurich DT (2014). Review of models used in economic analyses of new oral treatments for type 2 diabetes mellitus. Pharmacoeconomics.

[30] Becker C, Langer A, Leidl R (2011). The quality of three decision-analytic diabetes models: a systematic health economic assessment. Expert Rev Pharmacoecon Outcomes Res.

[31] Yi Y, Philips Z, Bergman G, Burslem K (2010). Economic models in type 2 diabetes. Curr Med Res Opin.

[32] Tarride JE, Hopkins R, Blackhouse G (2010). A review of methods used in long-term cost-effectiveness models of diabetes mellitus treatment. Pharmacoeconomics.

[33] Lerner DJ, Kannel WB (1986). Patterns of coronary heart disease morbidity and mortality in the sexes: a 26-year follow-up of the Framingham population. Am Heart J.

[34] Klein R, Klein BE, Moss SE (1993). Prevalence of microalbuminuria in older-onset diabetes. Diabetes Care.

[35] Ballard DJ, Humphrey LL, Melton LJ (1988). Epidemiology of persistent proteinuria in type II diabetes mellitus Population-based study in Rochester Minnesota. Diabetes.

[36] Javitt JC, Aiello LP, Chiang Y (1994). Preventive eye care in people with diabetes is cost-saving to the federal government. Implications for health-care reform. Diabetes Care.

[37] Klein R, Klein BE, Moss SE, Davis MD, DeMets DL (1989). The Wisconsin Epidemiologic Study of Diabetic Retinopathy. X. Four-year incidence and progression of diabetic retinopathy when age at diagnosis is 30 years or more. Arch Ophthalmol.

[38] Park JY, Kim HK, Chung YE, Kim SW, Hong SK, Lee KU (1998). Incidence and determinants of microalbuminuria in Koreans with type 2 diabetes. Diabetes Care.

[39] Dyck PJ, Kratz KM, Karnes JL (1993). The prevalence by staged severity of various types of diabetic neuropathy, retinopathy, and nephropathy in a population-based cohort: the Rochester Diabetic Neuropathy Study. Neurology.

[40] Weinstein MC, Coxson PG, Williams LW (1987). Forecasting coronary heart disease incidence, mortality, and cost: the Coronary Heart Disease Policy Model. Am J Public Health.

[41] Anderson KM, Odell PM, Wilson PW, Kannel WB (1991). Cardiovascular disease risk profiles. Am Heart J.

[42] Hunink MG, Goldman L, Tosteson AN (1997). The recent decline in mortality from coronary heart disease, 1980–1990. The effect of secular trends in risk factors and treatment. JAMA.

[43] Eastman RC, Javitt JC, Herman WH (1997). Model of complications of NIDDM. II. Analysis of the health benefits and cost-effectiveness of treating NIDDM with the goal of normoglycemia. Diabetes Care.

[44] D'Agostino RB, Russell MW, Huse DM (2000). Primary and subsequent coronary risk appraisal: new results from the Framingham study. Am Heart J.

[45] Herlitz J, Bång A, Karlson BW (1996). Mortality, place and mode of death and reinfarction during a period of 5 years after acute myocardial infarction in diabetic and non-diabetic patients. Cardiology.

[46] Almbrand B, Johannesson M, Sjöstrand B, Malmberg K, Rydén L (2000). Cost-effectiveness of intense insulin treatment after acute myocardial infarction in patients with diabetes mellitus; results from the DIGAMI study. Eur Heart J.

[47] Wolfe RA, Ashby VB, Milford EL (1999). Comparison of mortality in all patients on dialysis, patients on dialysis awaiting transplantation, and recipients of a first cadaveric transplant. N Engl J Med.

[48] Klein R, Klein BE, Moss SE, Davis MD, DeMets DL (1989). The wisconsin epidemiologic study of diabetic retinopathy. IX. Four-year incidence and progression of diabetic retinopathy when age at diagnosis is less than 30 years. Arch Ophthalmol.

[49] Chaturvedi N, Sjolie AK, Stephenson JM (1998). Effect of lisinopril on progression of retinopathy in normotensive people with type 1 diabetes. The EUCLID Study Group. EURODIAB Controlled Trial of Lisinopril in Insulin-Dependent Diabetes Mellitus. Lancet.

[50] Stratton IM, Kohner EM, Aldington SJ (2001). UKPDS 50: risk factors for incidence and progression of retinopathy in Type II diabetes over 6 years from diagnosis. Diabetologia.

[51] Partanen J, Niskanen L, Lehtinen J (1995). Natural history of peripheral neuropathy in patients with non-insulin-dependent diabetes mellitus. N Engl J Med.

[52] The effect of intensive diabetes therapy on the development and progression of neuropathy The Diabetes Control and Complications Trial Research Group (1995). Ann Intern Med 122(8):561–568. 10.7326/0003-4819-122-8-199504150-00001 10.7326/0003-4819-122-8-199504150-00001 7887548

[53] Petty GW, Brown RD, Whisnant JP (1998). Survival and recurrence after first cerebral infarction: a population-based study in Rochester, Minnesota, 1975 through 1989. Neurology.

[54] Sprafka JM, Virnig BA, Shahar E, McGovern PG (1994). Trends in diabetes prevalence among stroke patients and the effect of diabetes on stroke survival: the Minnesota Heart Survey. Diabet Med.

[55] Ragnarson Tennvall G, Apelqvist J (2001). Prevention of diabetes-related foot ulcers and amputations: a cost-utility analysis based on Markov model simulations. Diabetologia.

[56] Ben-Ami H, Nagachandran P, Mendelson A, Edoute Y (1999). Drug-induced hypoglycemic coma in 102 diabetic patients. Arch Intern Med.

[57] Stepka M, Rogala H, Czyzyk A (1993). Hypoglycemia: a major problem in the management of diabetes in the elderly. Aging (Milano).

[58] Intensive blood-glucose control with sulphonylureas or insulin compared with conventional treatment and risk of complications in patients with type 2 diabetes (UKPDS 33). UK Prospective Diabetes Study (UKPDS) Group (1998). Lancet 352(9131):837–853 9742976

[59] Malmberg K, Yusuf S, Gerstein HC (2000). Impact of diabetes on long-term prognosis in patients with unstable angina and non-Q-wave myocardial infarction: results of the OASIS (Organization to Assess Strategies for Ischemic Syndromes) Registry. Circulation.

[60] Ulvenstam G, Aberg A, Bergstrand R (1985). Long-term prognosis after myocardial infarction in men with diabetes. Diabetes.

[61] Löwel H, Koenig W, Engel S, Hörmann A, Keil U (2000). The impact of diabetes mellitus on survival after myocardial infarction: can it be modified by drug treatment? Results of a population-based myocardial infarction register follow-up study. Diabetologia.

[62] Stevens RJ, Kothari V, Adler AI, Stratton IM (2001). The UKPDS risk engine: a model for the risk of coronary heart disease in type II diabetes (UKPDS 56). Clin Sci (Lond).

[63] Gall MA, Hougaard P, Borch-Johnsen K, Parving HH (1997). Risk factors for development of incipient and overt diabetic nephropathy in patients with non-insulin dependent diabetes mellitus: prospective, observational study. BMJ.

[64] Ravid M, Savin H, Jutrin I (1993). Long-term stabilizing effect of angiotensin-converting enzyme inhibition on plasma creatinine and on proteinuria in normotensive type II diabetic patients. Ann Intern Med.

[65] Klein R, Klein BE, Moss SE, Cruickshanks KJ (1994). The Wisconsin Epidemiologic Study of diabetic retinopathy XIV Ten-year incidence and progression of diabetic retinopathy. Arch Ophthalmol.

[66] Klein R, Klein BE, Moss SE, Cruickshanks KJ (1995). The wisconsin epidemiologic study of diabetic retinopathy. XV. The long-term incidence of macular edema. Ophthalmology.

[67] Moss SE, Klein R, Klein BE (1988). The incidence of vision loss in a diabetic population. Ophthalmology.

[68] Moss SE, Klein R, Klein BE (1994). Ten-year incidence of visual loss in a diabetic population. Ophthalmology.

[69] Sands ML, Shetterly SM, Franklin GM, Hamman RF (1997). Incidence of distal symmetric (sensory) neuropathy in NIDDM. The San Luis valley diabetes study. Diabetes Care.

[70] Adler AI, Boyko EJ, Ahroni JH, Smith DG (1999). Lower-extremity amputation in diabetes. The independent effects of peripheral vascular disease, sensory neuropathy, and foot ulcers. Diabetes Care.

[71] Hier DB, Foulkes MA, Swiontoniowski M (1991). Stroke recurrence within 2 years after ischemic infarction. Stroke.

[72] Sacco RL, Shi T, Zamanillo MC, Kargman DE (1994). Predictors of mortality and recurrence after hospitalized cerebral infarction in an urban community: the Northern Manhattan Stroke Study. Neurology.

[73] Kothari V, Stevens RJ, Adler AI (2002). UKPDS 60: risk of stroke in type 2 diabetes estimated by the UK prospective diabetes study risk engine. Stroke.

[74] Currie CJ, Morgan CL, Peters JR (1998). The epidemiology and cost of inpatient care for peripheral vascular disease, infection, neuropathy, and ulceration in diabetes. Diabetes Care.

[75] Clarke P, Gray A, Legood R, Briggs A, Holman R (2003). The impact of diabetes-related complications on healthcare costs: results from the United Kingdom Prospective Diabetes Study (UKPDS Study No. 65). Diabet Med.

[76] Effect of intensive therapy on the development and progression of diabetic nephropathy in the Diabetes Control and Complications Trial. The Diabetes Control and Complications (DCCT) Research Group (1995). Kidney Int 47(6):1703–1720. 10.1038/ki.1995.236 10.1038/ki.1995.236 7643540

[77] Bagust A, Hopkinson PK, Maier W, Currie CJ (2001). An economic model of the long-term health care burden of type II diabetes. Diabetologia.

[78] Ahmad Kiadaliri A, Gerdtham UG, Nilsson P (2013). Towards renewed health economic simulation of type 2 diabetes: risk equations for first and second cardiovascular events from Swedish register data. PLoS ONE.

[79] Brown JB, Russell A, Chan W, Pedula K, Aickin M (2000). The global diabetes model: user friendly version 3.0. Diabetes Res Clin Pract.

[80] Gerstein HC, Miller ME, Genuth S (2011). Long-term effects of intensive glucose lowering on cardiovascular outcomes. N Engl J Med.

[81] Wing RR, Bolin P, Brancati FL (2013). Cardiovascular effects of intensive lifestyle intervention in type 2 diabetes. N Engl J Med.

[82] Perreault L, Pan Q, Mather KJ (2012). Effect of regression from prediabetes to normal glucose regulation on long-term reduction in diabetes risk: results from the diabetes prevention program outcomes study. Lancet.

[83] Patel A, MacMahon S, Chalmers J (2008). Intensive blood glucose control and vascular outcomes in patients with type 2 diabetes. N Engl J Med.

[84] Clarke PM, Simon J, Cull CA, Holman RR (2006). Assessing the impact of visual acuity on quality of life in individuals with type 2 diabetes using the short form-36. Diabetes Care.

[85] McEwan P, Bennett H, Ward T, Bergenheim K (2015). Refitting of the UKPDS 68 risk equations to contemporary routine clinical practice data in the UK. Pharmacoeconomics.

[86] Diabetes Control and Complications Trial Research Group (1995) The effect of intensive diabetes treatment on the progression of diabetic retinopathy in insulin-dependent diabetes mellitus. The Diabetes Control and Complications Trial. Arch Ophthalmol 113(1):36–51. 10.1001/archopht.1995.01100010038019 10.1001/archopht.1995.01100010038019 7826293

