# Supplementary material for: Decision models in type 2 diabetes mellitus: A systematic review
Source: Acta Diabetol. 2021 Jun 3;58(11):1451–69. doi: 10.1007/s00592-021-01742-6 (PMC8505393; doi:10.1007/s00592-021-01742-6)
Supplement: Supplementary file 1 — Supplementary file1 (DOCX 170 kb) [file 592_2021_1742_MOESM1_ESM.docx]

**Appendix 1 database search strategies**

| **Searches** | **Search Terms** |
| --- | --- |
| 1 | Diabetes Mellitus, Type 2 [MESH] |
| 2 | Diabetes Mellitus, Type 2 [Title/Abstract] |
| 3 | Non-Insulin-Dependent Diabetes Mellitus [Title/Abstract] |
| 4 | Stable Diabetes Mellitus [Title/Abstract] |
| 5 | Diabetes Mellitus, Type II [Title/Abstract] |
| 6 | NIDDM [Title/Abstract] |
| 7 | Maturity-Onset Diabetes Mellitus [Title/Abstract] |
| 8 | MODY [Title/Abstract] |
| 9 | Type 2 Diabetes Mellitus [Title/Abstract] |
| 10 | Maturity Onset Diabetes [Title/Abstract] |
| 11 | Type 2 Diabetes [Title/Abstract] |
| 12 | Diabetes, Type 2 [Title/Abstract] |
| 13 | Adult-Onset Diabetes Mellitus [Title/Abstract] |
| 14 | type II DM [Title/Abstract] |
| 15 | type 2 DM [Title/Abstract] |
| 16 | type II diabetes [Title/Abstract] |
| 17 | type II diabetic [Title/Abstract] |
| 18 | type 2 diabetic [Title/Abstract] |
| 19 | diabetes type II [Title/Abstract] |
| 20 | 1 or 2 or 3 or 4 or 5 or 6 or 7 or 8 or 9 or 10 or 11 or 12 or 13 or 14 or 15 or 16 or 17 or 18 or 19 |
| 21 | simulation model$ [Title/Abstract] |
| 22 | Markov [Title/Abstract] |
| 23 | monte carlo [Title/Abstract] |
| 24 | decision tree$ [Title/Abstract] |
| 25 | decision analy$ [Title/Abstract] |
| 26 | qaly$ [Title/Abstract] |
| 27 | utility value$ [Title/Abstract] |
| 28 | hui$1 [Title/Abstract] |
| 29 | qwb [Title/Abstract] |
| 30 | (qald$ or qale$ or qtime$) [Title/Abstract] |
| 31 | (well being or wellbeing) [Title/Abstract] |
| 32 | (daly or qol or hql or hqol or hrqol or hr ql or hrql) [Title/Abstract] |
| 33 | cost-utility [Title/Abstract] |
| 34 | cost-effectiveness [Title/Abstract] |
| 35 | cost-benefit [Title/Abstract] |
| 36 | cost-minimisation [Title/Abstract] |
| 37 | cost-minimization [Title/Abstract] |
| 38 | Modelling [Title/Abstract] |
| 39 | Modeling [Title/Abstract] |
| 40 | decision model [Title/Abstract] |
| 41 | QALY [Title/Abstract] |
| 42 | quality adjusted life year$ [Title/Abstract] |
| 43 | Cost [Title/Abstract] |
| 44 | life year$ [Title/Abstract] |
| 45 | incremental cost-effectiveness ratio [Title/Abstract] |
| 46 | (qtwist or q twist) [Title/Abstract] |
| 47 | 21 or 22 or 23 or 24 or 25 or 26 or 27 or 28 or 29 or 30 or 31 or 32 or 33 or 34 or 35 or 36 or 37 or 39 or 40 or 41 or 42  or 43 or 44 or 45 or 46 |
| 48 | 20 and 47 |

**Appendix 2 Checklist of information to extract for each identified model**

| **Parameter** | **Inclusion/exclusion criteria** |
| --- | --- |
| Basic Information | Title  First author  Country of publication  Year published  Language  Citation  Others |
| Study details | Model name  Study objective  Type of evaluation  Inclusion/exclusion criteria  Intervention and comparator details  Author’s conclusions and comments |
| Population characteristics | Mean/median age  Gender percentages  Ethnicity (percent)  Body mass index(BMI) |
| Basic modelling methodologies | Perspective (healthcare payer, societal)  Model type  Simulation method (cohort, patient-level)  Capture 2nd order (parameter) uncertainty  Cycle length  Time horizon  Discounting |
| Model structure (and key data sources/risk equations) | Baseline characteristics  Adverse events included  T2DM complications included and excluded  Risk factors included  Incorporation of baseline risk of T2DM complications  Incorporation of treatment effects |
| Data inputs for the included applications | Sources and availability (baseline risk, efficacy, quality of life, resource use) |
| Model outcomes | Health utility outcomes (e.g., QALYs, LYGs)  Other outcomes (e.g., life years and diabetes-related events) |
| Model validation | Face validation procedures  Internal validation procedures  External validation procedures  Cross-validation procedures  Predictive validation procedures |
| Model uncertainty | One-way sensitivity analysis  Multi-way sensitivity analysis  Probabilistic sensitivity analysis(PSA) |

**Appendix 3 Summary of secondary publications associated with the models included in the review**

| Model | Primary publication | Secondary publication |
| --- | --- | --- |
| The CDM model | Palmer AJ 2004 | Foos, V. 2019[1]  Bilir, S. P. 2018[2]  Viljoen, A. 2019[3]  Roze, S. 2019[4]  Raya, P. M. 2019[5]  Pohlmann, J. 2019[6]  Malkin, S. J. P. 2019[7]  Gorgojo-Martinez, J. J. 2020[8]  Su, W. 2019[9]  Ramos, M. 2019[10]  Pohlmann, J. 2019[11]  Malkin, S. J. P. 2019[12]  Lau, E. 2019[13]  Hunt, B 2019[14]  Gaede, P. 2019[15]  Gaede, P. 2019[16]  Bain, S. C. 2019[17]  Bain, S. C. 2020[18]  Barnett, A. H. 2018[19]  Pawaskar, M. 2019[20]  Ishii, H. 2018[21]  Cho, J. H. 2008[22]  Pollock, R. F. 2013[23]  Basson, M. 2018[24]  Vega-Hernandez, G. 2017[25]  Valentine, W. J. 2007[26]  Yang, L. 2012[27]  Valentine, W. J. 2007[28]  Ray, J. A. 2007[29]  Ray, J. A. 2007[30]  Roze, S. 2006[31]  Fonda, S. J. 2016[32]  Marsh, K. 2016[33]  Psota, M. 2017[34]  Tunis, S. L. 2009[35]  Permsuwan, U. 2017[36]  Mezquita-Raya, P. 2017[37]  Kvapil, M. 2017[38]  Hunt, B. 2017[39]  Hunt, B. 2017[40]  Zhang, X. 2016[41]  Schlueter, M. 2016[42]  Permsuwan, U. 2016[43]  Brown, S. 2016[44]  Perez, A. 2015[45]  Home, P. 2015[46]  Shafie, A. A. 2014[47]  Brown, S. T. 2014[48]  Mezquita Raya, P. 2013[49]  Valov, V. 2012[50]  Samyshkin, Y. 2012[51]  Pollock, R. F. 2012[52]  Pollock, R. F. 2012[53]  Lee, W. C. 2012[54]  Guillermin, A. L. 2012[55]  Davies, M. J. 2012[56]  Valentine, W. J. 2011[57]  Lee, W. C. 2011[58]  Palmer, J. L. 2010[59]  Palmer, J. L. 2010[60]  Sullivan, S. D. 2009[61]  Sullivan, S. D. 2009[62]  Valentine, W. J. 2009[63]  Scherbaum, W. A. 2009[64]  St. Charles, M. 2009[65]  Lee, K. H. 2009[66]  Mittendorf, T. 2009[67]  Goodall, G. 2009[68]  Cameron, C. G. 2009[69]  Tunis, S. L. 2008[70]  Palmer, J. L. 2008[71]  Ray, J. A. 2008[72]  Palmer, J. L. 2008[73]  Goodall, G. 2008[74]  Minshall, M. E. 2008[75]  Chirakup, S. 2008[76]  Ali, M. 2008[77]  Men, P. 2020[78] |
| the UKPDS-OM1 model | Clarke, P. M. 2004 | Gilmer, T. 2018[79]  Varney, J. E. 2016[80]  Odnoletkova, I. 2016[81]  Tao, L. 2015[82]  Staff, M. 2015[83]  Cameron, C. 2010[84]  McRae, I. S. 2008[85]  Kousoulakou, H. 2017[86]  Granström, O. 2012[87]  Pollock, R. F. 2011[88]  Klarenbach, S. 2011[89]  Gao, L. 2012[90] |
| the UKPDS-OM2 model | Hayes, A. J. 2013 | Nerat, T. 2016[91]  Mostafa, S. A. 2018[92]  Pollock, R. F. 2019[93] |
| the Archimedes model | David M Eddy 2003 | Gray, B.2012[94]  Dziuba, J. 2014[95]  Gaebler, J. A. 2012[96]  Peskin, B. R. 2011[97] |
| the Cardiff model | McEwan, P. 2006 | Cai, X. 2019[98]  Capel, M. 2018[99]  Gu, S. Y. 2017[100]  Tzanetakos, C. 2016[101]  Tzanetakos, C. 2016[102]  Gu, S. 2016[103]  Gu, S. 2016[104]  Gordon, J. 2016[105]  Chuang, L. H. 2016[106]  Sabale, U. 2015[107]  Charokopou, M. 2015[108]  Deng, J. 2015[109]  Charokopou, M. 2015[110]  van Haalen, H. G. 2014[111]  Grzeszczak, W. 2012[112]  Erhardt, W. 2012[113] |
| the COMT model | Wu, B. 2018 | Hou, X. Y. 2019[114] |
| the ECHO model | Willis, M. 2013 | Gupta, V. 2019[115]  Neslusan, C. 2018[116] |
| the IHE model | Lundqvist, A. 2014 | Johansen, P. 2019[117]  Ericsson, A. 2018[118]  Steen Carlsson, K 2014[119] |
| the Michigan model | Zhou, H. 2005 | Kuo, S. 2018[120]  Herman, W. H. 2015[121] |
| the DCEM model | Hoerger, T. J. 2002 | Ryabov, I. 2014[122]  Shao, H. 2019[123] |
| The NIDDM model | Eastman, R. C. 1997 | Eastman, R. C. 1997[124] |
| the Sheffield model | Gillett, M. 2010 | Gillett, M. 2015[125] |
| the CDS model | Zhuo T. Su 2019 | **—** |
| the ODEM model | O'Reilly, D. 2007 | O'Reilly, D. 2012[126] |

**Reference：**

1. Foos V, Wang K, McEwan P, Zhang Y, Xin P, Jiang X, Qu S, Xiong T, De Moor R, Ramos M, Lamotte M, Ji L (2018) Assessing the Burden of Type 2 Diabetes in China Considering the Current Status-Quo Management and Implications of Improved Management Using a Modeling Approach. Value Health Reg Issues 18:36-46. doi:10.1016/j.vhri.2018.08.006

2. Bilir SP, Hellmund R, Wehler E, Li H, Munakata J, Lamotte M (2018) The Cost-effectiveness of a Flash Glucose Monitoring System for Management of Patients with Type 2 Diabetes Receiving Intensive Insulin Treatment in Sweden. Eur Endocrinol 14 (2):80-85. doi:10.17925/ee.2018.14.2.80

3. Viljoen A, Hoxer CS, Johansen P, Malkin S, Hunt B, Bain SC (2019) Evaluation of the long-term cost-effectiveness of once-weekly semaglutide versus dulaglutide for treatment of type 2 diabetes mellitus in the UK. Diabetes Obes Metab 21 (3):611-621. doi:10.1111/dom.13564

4. Roze S, Smith-Palmer J, Delbaere A, Bjornstrom K, de Portu S, Valentine W, Honkasalo M (2019) Cost-Effectiveness of Continuous Subcutaneous Insulin Infusion Versus Multiple Daily Injections in Patients with Poorly Controlled Type 2 Diabetes in Finland. Diabetes Ther 10 (2):563-574. doi:10.1007/s13300-019-0575-9

5. Raya PM, Blasco FJA, Hunt B, Martin V, Thorsted BL, Basse A, Price H (2019) Evaluating the long-term cost-effectiveness of fixed-ratio combination insulin degludec/liraglutide (IDegLira) for type 2 diabetes in Spain based on real-world clinical evidence. Diabetes Obes Metab 21 (6):1349-1356. doi:10.1111/dom.13660

6. Pohlmann J, Russel-Szymczyk M, Holik P, Rychna K, Hunt B (2019) Treating Patients with Type 2 Diabetes Mellitus Uncontrolled on Basal Insulin in the Czech Republic: Cost-Effectiveness of IDegLira Versus iGlarLixi. Diabetes Ther 10 (2):493-508. doi:10.1007/s13300-019-0569-7

7. Malkin SJP, Russel-Szymczyk M, Liidemann G, Volke V, Hunt B (2019) Once-Weekly Semaglutide Versus Once-Daily Liraglutide for the Treatment of Type 2 Diabetes: A Long-Term Cost-Effectiveness Analysis in Estonia. Diabetes Ther 10 (1):159-176. doi:10.1007/s13300-018-0542-x

8. Gorgojo-Martinez JJ, Malkin SJP, Martin V, Hallen N, Hunt B (2020) Assessing the cost-effectiveness of a once-weekly GLP-1 analogue versus an SGLT-2 inhibitor in the Spanish setting: Once-weekly semaglutide versus empagliflozin. Journal of Medical Economics 23 (2):193-203. doi:10.1080/13696998.2019.1681436

9. Su W, Li CY, Zhang L, Lin ZY, Tan J, Xuan JW (2019) Meta-Analysis and Cost-Effectiveness Analysis of Insulin Glargine 100 U/mL Versus Insulin Degludec for the Treatment of Type 2 Diabetes in China. Diabetes Therapy 10 (5):1969-1984. doi:10.1007/s13300-019-00683-2

10. Ramos M, Foos V, Ustyugova A, Hau N, Gandhi P, Lamotte M (2019) Cost-Effectiveness Analysis of Empagliflozin in Comparison to Sitagliptin and Saxagliptin Based on Cardiovascular Outcome Trials in Patients with Type 2 Diabetes and Established Cardiovascular Disease. Diabetes Therapy 10 (6):2153-2167. doi:10.1007/s13300-019-00701-3

11. Pöhlmann J, Montagnoli R, Lastoria G, Parekh W, Markert M, Hunt B (2019) Value for money in the treatment of patients with type 2 diabetes mellitus: Assessing the long-term cost-effectiveness of ideglira versus iGlarLixi in Italy. ClinicoEconomics and Outcomes Research 11:605-614. doi:10.2147/CEOR.S218746

12. Malkin SJP, Russel-Szymczyk M, Psota M, Hlavinkova L, Hunt B (2019) The Management of Type 2 Diabetes with Once-Weekly Semaglutide Versus Dulaglutide: A Long-Term Cost-Effectiveness Analysis in Slovakia. Advances in Therapy 36 (8):2034-2051. doi:10.1007/s12325-019-00965-y

13. Lau E, Salem A, Chan JCN, So WY, Kong A, Lamotte M, Luk A (2019) Insulin glargine compared to neutral protamine Hagedorn (NPH) insulin in patients with type-2 diabetes uncontrolled with oral anti-diabetic agents alone in Hong Kong: a cost-effectiveness analysis. Cost Eff Resour Alloc 17:13. doi:10.1186/s12962-019-0180-9

14. Hunt B, Malkin SJP, Moes RGJ, Huisman EL, Vandebrouck T, Wolffenbuttel B (2019) Once-weekly semaglutide for patients with type 2 diabetes: A cost-effectiveness analysis in the Netherlands. BMJ Open Diabetes Research and Care 7 (1). doi:10.1136/bmjdrc-2019-000705

15. Gaede P, Johansen P, Tikkanen CK, Pollock RF, Hunt B, Malkin SJP (2019) Management of Patients with Type 2 Diabetes with Once-Weekly Semaglutide Versus Dulaglutide, Exenatide ER, Liraglutide and Lixisenatide: A Cost-Effectiveness Analysis in the Danish Setting. Diabetes Therapy 10 (4):1297-1317. doi:10.1007/s13300-019-0630-6

16. Gæde P, Johansen P, Tikkanen CK, Pollock RF, Hunt B, Malkin SJP (2019) Correction to: Management of Patients with Type 2 Diabetes with Once-Weekly Semaglutide Versus Dulaglutide, Exenatide ER, Liraglutide and Lixisenatide: A Cost-Effectiveness Analysis in the Danish Setting (Diabetes Therapy, (2019), 10, 4, (1297-1317), 10.1007/s13300-019-0630-6). Diabetes Therapy 10 (4):1319-1321. doi:10.1007/s13300-019-0638-y

17. Bain SC, Hansen BB, Malkin SJP, Nuhoho S, Valentine WJ, Chubb B, Hunt B, Capehorn M (2019) Oral Semaglutide Versus Empagliflozin, Sitagliptin and Liraglutide in the UK: Long-Term Cost-Effectiveness Analyses Based on the PIONEER Clinical Trial Programme. Diabetes Therapy. doi:10.1007/s13300-019-00736-6

18. Bain SC, Bekker Hansen B, Hunt B, Chubb B, Valentine WJ (2020) Evaluating the burden of poor glycemic control associated with therapeutic inertia in patients with type 2 diabetes in the UK. J Med Econ 23 (1):98-105. doi:10.1080/13696998.2019.1645018

19. Barnett AH, Arnoldini S, Hunt B, Subramanian G, Hoxer CS (2018) Switching from sitagliptin to liraglutide to manage patients with type 2 diabetes in the UK: A long-term cost-effectiveness analysis. Diabetes Obes Metab 20 (8):1921-1927. doi:10.1111/dom.13318

20. Pawaskar M, Bilir SP, Kowal S, Gonzalez C, Rajpathak S, Davies G (2019) Cost-effectiveness of intensification with sodium-glucose co-transporter-2 inhibitors in patients with type 2 diabetes on metformin and sitagliptin vs direct intensification with insulin in the United Kingdom. Diabetes, Obesity and Metabolism 21 (4):1010-1017. doi:10.1111/dom.13618

21. Ishii H, Madin-Warburton M, Strizek A, Thornton-Jones L, Suzuki S (2018) The cost-effectiveness of dulaglutide versus insulin glargine for the treatment of type 2 diabetes mellitus in Japan. J Med Econ 21 (5):488-496. doi:10.1080/13696998.2018.1431918

22. Cho JH, Lee JH, Oh JA, Kang MJ, Choi YH, Kwon HS, Chang SA, Cha BY, Son HY, Yoon KH (2008) Complication reducing effect of the information technology-based diabetes management system on subjects with type 2 diabetes. J Diabetes Sci Technol 2 (1):76-81. doi:10.1177/193229680800200111

23. Pollock RF, Muduma G, Valentine WJ (2013) Evaluating the cost-effectiveness of laparoscopic adjustable gastric banding versus standard medical management in obese patients with type 2 diabetes in the UK. Diabetes Obes Metab 15 (2):121-129. doi:10.1111/j.1463-1326.2012.01692.x

24. Basson M, Ntais D, Ayyub R, Wright D, Lowin J, Chartier F, Roze S, Norrbacka K (2018) The Cost-effectiveness of Dulaglutide 1.5mg versus Exenatide QW for the Treatment of Patients with Type 2 Diabetes Mellitus in France. Diabetes Ther 9 (1):13-25. doi:10.1007/s13300-017-0321-0

25. Vega-Hernandez G, Wojcik R, Schlueter M (2017) Cost-Effectiveness of Liraglutide Versus Dapagliflozin for the Treatment of Patients with Type 2 Diabetes Mellitus in the UK. Diabetes Ther 8 (3):513-530. doi:10.1007/s13300-017-0250-y

26. Valentine WJ, Erny-Albrecht KM, Ray JA, Roze S, Cobden D, Palmer AJ (2007) Therapy conversion to insulin detemir among patients with type 2 diabetes treated with oral agents: a modeling study of cost-effectiveness in the United States. Adv Ther 24 (2):273-290

27. Yang L, Christensen T, Sun FY, Chang JH (2012) Cost-Effectiveness of Switching Patients with Type 2 Diabetes from Insulin Glargine to Insulin Detemir in Chinese Setting: A Health Economic Model Based on the PREDICTIVE Study. Value in Health 15 (1):S56-S59. doi:10.1016/j.jval.2011.11.018

28. Valentine WJ, Bottomley JM, Palmer AJ, Brändle M, Foos V, Williams R, Dormandy JA, Yates J, Tan MH, Massi-Benedetti M (2007) PROactive 06: Cost-effectiveness of pioglitazone in Type 2 diabetes in the UK. Diabetic Medicine 24 (9):982-1002. doi:10.1111/j.1464-5491.2007.02188.x

29. Ray JA, Valentine WJ, Roze S, Nicklasson L, Cobden D, Raskin P, Garber A, Palmer AJ (2007) Insulin therapy in type 2 diabetes patients failing oral agents: cost-effectiveness of biphasic insulin aspart 70/30 vs. insulin glargine in the US. Diabetes Obes Metab 9 (1):103-113. doi:10.1111/j.1463-1326.2006.00581.x

30. Ray JA, Boye KS, Yurgin N, Valentine WJ, Roze S, McKendrick J, Tucker DM, Foos V, Palmer AJ (2007) Exenatide versus insulin glargine in patients with type 2 diabetes in the UK: a model of long-term clinical and cost outcomes. Curr Med Res Opin 23 (3):609-622. doi:10.1185/030079907x178685

31. Roze S, Valentine WJ, Evers T, Palmer AJ (2006) Acarbose in addition to existing treatments in patients with type 2 diabetes: Health economic analysis in a German setting. Current Medical Research and Opinion 22 (7):1415-1424. doi:10.1185/030079906X115531

32. Fonda SJ, Graham C, Munakata J, Powers JM, Price D, Vigersky RA (2016) The Cost-Effectiveness of Real-Time Continuous Glucose Monitoring (RT-CGM) in Type 2 Diabetes. J Diabetes Sci Technol 10 (4):898-904. doi:10.1177/1932296816628547

33. Marsh K, Ganz M, Nortoft E, Lund N, Graff-Zivin J (2016) Incorporating environmental outcomes into a health economic model. International Journal of Technology Assessment in Health Care 32 (6):400-406. doi:10.1017/S0266462316000581

34. Psota M, Psenkova MB, Racekova N, Ramirez de Arellano A, Vandebrouck T, Hunt B (2017) Cost-effectiveness analysis of IDegLira versus basal-bolus insulin for patients with type 2 diabetes in the Slovak health system. Clinicoecon Outcomes Res 9:749-762. doi:10.2147/ceor.S143127

35. Tunis SL (2009) A cost-effectiveness analysis to illustrate the impact of cost definitions on results, interpretations and comparability of pharmacoeconomic studies in the US. Pharmacoeconomics 27 (9):735-744. doi:10.2165/10899600-000000000-00000

36.Permsuwan U, Thavorn K, Dilokthornsakul P, Saokaew S, Chaiyakunapruk N (2017) Cost-effectiveness of insulin detemir versus insulin glargine for Thai type 2 diabetes from a payer's perspective. J Med Econ 20 (9):991-999. doi:10.1080/13696998.2017.1347792

37. Mezquita-Raya P, Ramirez de Arellano A, Kragh N, Vega-Hernandez G, Pohlmann J, Valentine WJ, Hunt B (2017) Liraglutide Versus Lixisenatide: Long-Term Cost-Effectiveness of GLP-1 Receptor Agonist Therapy for the Treatment of Type 2 Diabetes in Spain. Diabetes Ther 8 (2):401-415. doi:10.1007/s13300-017-0239-6

38. Kvapil M, Prazny M, Holik P, Rychna K, Hunt B (2017) Cost-Effectiveness of IDegLira Versus Insulin Intensification Regimens for the Treatment of Adults with Type 2 Diabetes in the Czech Republic. Diabetes Ther 8 (6):1331-1347. doi:10.1007/s13300-017-0323-y

39. Hunt B, Ye Q, Valentine WJ, Ashley D (2017) Evaluating the Long-Term Cost-Effectiveness of Daily Administered GLP-1 Receptor Agonists for the Treatment of Type 2 Diabetes in the United Kingdom. Diabetes Ther 8 (1):129-147. doi:10.1007/s13300-016-0219-2

40. Hunt B, Glah D, van der Vliet M (2017) Modeling the Long-Term Cost-Effectiveness of IDegLira in Patients with Type 2 Diabetes Who are Failing To Meet Glycemic Targets on Basal Insulin Alone in The Netherlands. Diabetes Ther 8 (4):753-765. doi:10.1007/s13300-017-0266-3

41. Zhang X, Liu S, Li Y, Wang Y, Tian M, Liu G (2016) Long-Term Effectiveness and Cost-Effectiveness of Metformin Combined with Liraglutide or Exenatide for Type 2 Diabetes Mellitus Based on the CORE Diabetes Model Study. PLoS One 11 (6):e0156393. doi:10.1371/journal.pone.0156393

42. Schlueter M, Vega-Hernandez G, Wojcik R (2016) Cost-effectiveness of liraglutide versus dapagliflozin for the treatment of patients with type-2 diabetes mellitus in the UK. Value in Health 19 (7):A675

43.Permsuwan U, Dilokthornsakul P, Saokaew S, Thavorn K, Chaiyakunapruk N (2016) Cost-effectiveness of dipeptidyl peptidase-4 inhibitor monotherapy in elderly type 2 diabetes patients in Thailand. Clinicoecon Outcomes Res 8:521-529. doi:10.2147/ceor.S113559

44. Brown S, Al Hamarneh YN, Tsuyuki RT, Nehme K, Sauriol L (2016) Economic analysis of insulin initiation by pharmacists in a Canadian setting: The RxING study. Canadian Pharmacists Journal 149 (3):130-137. doi:10.1177/1715163516640813

45. Perez A, Mezquita Raya P, Ramirez de Arellano A, Briones T, Hunt B, Valentine WJ (2015) Cost-Effectiveness Analysis of Incretin Therapy for Type 2 Diabetes in Spain: 1.8 mg Liraglutide Versus Sitagliptin. Diabetes Ther 6 (1):61-74. doi:10.1007/s13300-015-0103-5

46. Home P, Baik SH, Gálvez GG, Malek R, Nikolajsen A (2015) An analysis of the cost-effectiveness of starting insulin detemir in insulin-naïve people with type 2 diabetes. Journal of Medical Economics 18 (3):230-240. doi:10.3111/13696998.2014.985788

47. Shafie AA, Gupta V, Baabbad R, Hammerby E, Home P (2014) An analysis of the short- and long-term cost-effectiveness of starting biphasic insulin aspart 30 in insulin-naive people with poorly controlled type 2 diabetes. Diabetes Res Clin Pract 106 (2):319-327. doi:10.1016/j.diabres.2014.08.024

48. Brown ST, Sauriol L (2014) Cost-effectiveness of insulin glargine versus sitagliptin in insulin naïve patients with type 2 diabetes mellitus. Journal of Population Therapeutics and Clinical Pharmacology 21 (1):e132-e133

49. Mezquita Raya P, Perez A, Ramirez de Arellano A, Briones T, Hunt B, Valentine WJ (2013) Incretin therapy for type 2 diabetes in Spain: a cost-effectiveness analysis of liraglutide versus sitagliptin. Diabetes Ther 4 (2):417-430. doi:10.1007/s13300-013-0044-9

50. Valov V, Palmer J, Czech M, Savova A, Petrova G (2012) COST-EFFECTIVENESS OF BIPHASIC INSULIN ASPART 30 VS. HUMAN PREMIX INSULIN IN TYPE 2 DIABETES FROM THE PAYER'S PERSPECTIVE IN BULGARIA. Biotechnology & Biotechnological Equipment 26 (2):2937-2944. doi:10.5504/bbeq.2012.0015

51. Samyshkin Y, Guillermin AL, Best JH, Brunell SC, Lloyd A (2012) Long-term cost-utility analysis of exenatide once weekly versus insulin glargine for the treatment of type 2 diabetes patients in the US. J Med Econ 15 Suppl 2:6-13. doi:10.3111/13696998.2012.708691

52. Pollock RF, Curtis BH, Valentine WJ (2012) A long-term analysis evaluating the cost-effectiveness of biphasic insulin lispro mix 75/25 and mix 50/50 versus long-acting basal insulin analogs in the United States. J Med Econ 15 (4):766-775. doi:10.3111/13696998.2012.675890

53. Pollock RF, Curtis BH, Smith-Palmer J, Valentine WJ (2012) A UK analysis of the cost-effectiveness of Humalog Mix75/25 and Mix50/50 versus long-acting basal insulin. Adv Ther 29 (12):1051-1066. doi:10.1007/s12325-012-0065-1

54. Lee WC, Samyshkin Y, Langer J, Palmer JL (2012) Long-term clinical and economic outcomes associated with liraglutide versus sitagliptin therapy when added to metformin in the treatment of type 2 diabetes: a CORE Diabetes Model analysis. J Med Econ 15 Suppl 2:28-37. doi:10.3111/13696998.2012.716111

55.Guillermin AL, Lloyd A, Best JH, DeYoung MB, Samyshkin Y, Gaebler JA (2012) Long-term cost-consequence analysis of exenatide once weekly vs sitagliptin or pioglitazone for the treatment of type 2 diabetes patients in the United States. J Med Econ 15 (4):654-663. doi:10.3111/13696998.2012.670677

56. Davies MJ, Chubb BD, Smith IC, Valentine WJ (2012) Cost-utility analysis of liraglutide compared with sulphonylurea or sitagliptin, all as add-on to metformin monotherapy in Type 2 diabetes mellitus. Diabet Med 29 (3):313-320. doi:10.1111/j.1464-5491.2011.03429.x

57. Valentine WJ, Palmer AJ, Lammert M, Langer J, Brandle M (2011) Evaluating the long-term cost-effectiveness of liraglutide versus exenatide BID in patients with type 2 diabetes who fail to improve with oral antidiabetic agents. Clin Ther 33 (11):1698-1712. doi:10.1016/j.clinthera.2011.09.022

58. Lee WC, Conner C, Hammer M (2011) Cost-effectiveness of liraglutide versus rosiglitazone, both in combination with glimepiride in treatment of type 2 diabetes in the US. Curr Med Res Opin 27 (5):897-906. doi:10.1185/03007995.2011.559444

59. Palmer JL, Beaudet A, White J, Plun-Favreau J, Smith-Palmer J (2010) Cost-effectiveness of biphasic insulin aspart versus insulin glargine in patients with type 2 diabetes in China. Adv Ther 27 (11):814-827. doi:10.1007/s12325-010-0078-6

60. Palmer JL, Knudsen MS, Aagren M, Thomsen TL (2010) Cost-effectiveness of switching to biphasic insulin aspart from human premix insulin in a US setting. J Med Econ 13 (2):212-220. doi:10.3111/13696991003723999

61. Sullivan SD, Alfonso-Cristancho R, Conner C, Hammer M, Blonde L (2009) Long-term outcomes in patients with type 2 diabetes receiving glimepiride combined with liraglutide or rosiglitazone. Cardiovascular Diabetology 8. doi:10.1186/1475-2840-8-12

62. Sullivan SD, Alfonso-Cristancho R, Conner C, Hammer M, Blonde L (2009) A simulation of the comparative long-term effectiveness of liraglutide and glimepiride monotherapies in patients with type 2 diabetes mellitus. Pharmacotherapy 29 (11):1280-1288

63.Valentine WJ, Tucker D, Palmer AJ, Minshall ME, Foos V, Silberman C (2009) Long-term cost-effectiveness of pioglitazone versus placebo in addition to existing diabetes treatment: a US analysis based on PROactive. Value Health 12 (1):1-9. doi:10.1111/j.1524-4733.2008.00403.x

64. Scherbaum WA, Goodall G, Erny-Albrecht KM, Massi-Benedetti M, Erdmann E, Valentine WJ (2009) Cost-effectiveness of pioglitazone in type 2 diabetes patients with a history of macrovascular disease: a German perspective. Cost Eff Resour Alloc 7:9. doi:10.1186/1478-7547-7-9

65. St Charles M, Minshall ME, Pandya BJ, Baran RW, Tunis SL (2009) A cost-effectiveness analysis of pioglitazone plus metformin compared with rosiglitazone plus metformin from a third-party payer perspective in the US. Curr Med Res Opin 25 (6):1343-1353. doi:10.1185/03007990902870084

66. Lee KH, Seo SJ, Smith-Palmer J, Palmer JL, White J, Valentine WJ (2009) Cost-effectiveness of switching to biphasic insulin aspart 30 from human insulin in patients with poorly controlled type 2 diabetes in South Korea. Value Health 12 Suppl 3:S55-61. doi:10.1111/j.1524-4733.2009.00628.x

67. Mittendorf T, Smith-Palmer J, Timlin L, Happich M, Goodall G (2009) Evaluation of exenatide vs. insulin glargine in type 2 diabetes: cost-effectiveness analysis in the German setting. Diabetes Obes Metab 11 (11):1068-1079. doi:10.1111/j.1463-1326.2009.01099.x

68. Goodall G, Sarpong EM, Hayes C, Valentine WJ (2009) The consequences of delaying insulin initiation in UK type 2 diabetes patients failing oral hyperglycaemic agents: a modelling study. BMC Endocr Disord 9:19. doi:10.1186/1472-6823-9-19

69. Cameron CG, Bennett HA (2009) Cost-effectiveness of insulin analogues for diabetes mellitus. Cmaj 180 (4):400-407. doi:10.1503/cmaj.081180

70. Tunis SL, Minshall ME, St Charles M, Pandya BJ, Baran RW (2008) Pioglitazone versus rosiglitazone treatment in patients with type 2 diabetes and dyslipidemia: cost-effectiveness in the US. Curr Med Res Opin 24 (11):3085-3096. doi:10.1185/03007990802434874

71. Palmer JL, Goodall G, Nielsen S, Kotchie RW, Valentine WJ, Palmer AJ, Roze S (2008) Cost-effectiveness of insulin aspart versus human soluble insulin in type 2 diabetes in four European countries: subgroup analyses from the PREDICTIVE study. Curr Med Res Opin 24 (5):1417-1428. doi:10.1185/030079908x297295

72. Ray JA, Huet D, Valentine WJ, Palmer AJ, Cugnardey N, Renaudin C (2008) Long-term costs and clinical outcomes associated with metformin-glibenclamide combination tablets (Glucovance®) in patients with type 2 diabetes sub-optimally controlled by metformin: A modelling study in the French setting. British Journal of Diabetes and Vascular Disease 8 (1):39-44. doi:10.1177/14746514080080010901

73. Palmer JL, Gibbs M, Scheijbeler HW, Kotchie RW, Nielsen S, White J, Valentine WJ (2008) Cost-effectiveness of switching to biphasic insulin aspart in poorly-controlled type 2 diabetes patients in China. Adv Ther 25 (8):752-774. doi:10.1007/s12325-008-0080-4

74. Goodall G, Jendle JH, Valentine WJ, Munro V, Brandt AB, Ray JA, Roze S, Foos V, Palmer AJ (2008) Biphasic insulin aspart 70/30 vs. insulin glargine in insulin naïve type 2 diabetes patients: Modelling the long-term health economic implications in a Swedish setting. International Journal of Clinical Practice 62 (6):869-876. doi:10.1111/j.1742-1241.2008.01766.x

75. Minshall ME, Oglesby AK, Wintle ME, Valentine WJ, Roze S, Palmer AJ (2008) Estimating the long-term cost-effectiveness of exenatide in the United States: an adjunctive treatment for type 2 diabetes mellitus. Value Health 11 (1):22-33. doi:10.1111/j.1524-4733.2007.00211.x

76. Chirakup S, Chaiyakunapruk N, Chaikledkeaw U, Pongcharoensuk P, Ongphiphadhanakul B, Roze S, Valentine WJ, Palmer AJ (2008) Cost-effectiveness analysis of thiazolidinediones in uncontrolled type 2 diabetic patients receiving sulfonylureas and metformin in Thailand. Value Health 11 Suppl 1:S43-51. doi:10.1111/j.1524-4733.2008.00366.x

77. Ali M, White J, Lee CH, Palmer JL, Smith-Palmer J, Fakhoury W, Valentine WJ (2008) Therapy conversion to biphasic insulin aspart 30 improves long-term outcomes and reduces the costs of type 2 diabetes in Saudi Arabia. J Med Econ 11 (4):651-670. doi:10.3111/13696990802589122

78. Men P, Qu SL, Luo WT, Li CY, Zhai SD (2020) Comparison of lixisenatide in combination with basal insulin vs other insulin regimens for the treatment of patients with type 2 diabetes inadequately controlled by basal insulin: Systematic review, network meta-analysis and cost-effectiveness analysis. Diabetes Obesity & Metabolism 22 (1):107-115. doi:10.1111/dom.13871

79. Gilmer T, O'Connor PJ, Schiff JS, Taylor G, Vazquez-Benitez G, Garrett JE, Vue-Her H, Rinn S, Anderson J, Desai J (2018) Cost-Effectiveness of a Community-Based Diabetes Prevention Program with Participation Incentives for Medicaid Beneficiaries. Health Serv Res 53 (6):4704-4724. doi:10.1111/1475-6773.12973

80. Varney JE, Liew D, Weiland TJ, Inder WJ, Jelinek GA (2016) The cost-effectiveness of hospital-based telephone coaching for people with type 2 diabetes: a 10 year modelling analysis. BMC Health Serv Res 16 (1):521. doi:10.1186/s12913-016-1645-6

81. Odnoletkova I, Ramaekers D, Nobels F, Goderis G, Aertgeerts B, Annemans L (2016) Delivering Diabetes Education through Nurse-Led Telecoaching. Cost-Effectiveness Analysis. PLoS One 11 (10):e0163997. doi:10.1371/journal.pone.0163997

82. Tao L, Wilson ECF, Wareham NJ, Sandbæk A, Rutten GEHM, Lauritzen T, Khunti K, Davies MJ, Borch-Johnsen K, Griffin SJ, Simmons RK (2015) Cost-effectiveness of intensive multifactorial treatment compared with routine care for individuals with screendetected Type 2 diabetes: Analysis of the ADDITION-UK cluster-randomized controlled trial. Diabetic Medicine 32 (7):907-919. doi:10.1111/dme.12711

83. Staff M, Chen JS, March L (2015) Using computer modelled life expectancy to evaluate the impact of Australian Primary Care Incentive programs for patients with type 2 diabetes. Diabetes Res Clin Pract 109 (2):319-325. doi:10.1016/j.diabres.2015.05.012

84. Cameron C, Coyle D, Ur E, Klarenbach S (2010) Cost-effectiveness of self-monitoring of blood glucose in patients with type 2 diabetes mellitus managed without insulin. Cmaj 182 (1):28-34. doi:10.1503/cmaj.090765

85. McRae IS, Butler JRG, Sibthorpe BM, Ruscoe W, Snow J, Rubiano D, Gardner KL (2008) A cost effectiveness study of integrated care in health services delivery: A diabetes program in Australia. BMC Health Services Research 8. doi:10.1186/1472-6963-8-205

86. Kousoulakou H, Hatzikou M, Baroutsou V, Yfantopoulos J (2017) Cost effectiveness of vildagliptin versus glimepiride as add-on treatment to metformin for the treatment of diabetes mellitus type 2 patients in Greece. Cost Effectiveness and Resource Allocation 15 (1). doi:10.1186/s12962-017-0082-7

87. Granström O, Bergenheim K, McEwan P, Sennfält K, Henriksson M (2012) Cost-effectiveness of saxagliptin (Onglyza®) in type 2 diabetes in Sweden. Primary Care Diabetes 6 (2):127-136. doi:10.1016/j.pcd.2011.09.003

88. Pollock RF, Valentine WJ, Pilgaard T, Nishimura H (2011) The cost effectiveness of rapid-acting insulin aspart compared with human insulin in type 2 diabetes patients: an analysis from the Japanese third-party payer perspective. J Med Econ 14 (1):36-46. doi:10.3111/13696998.2010.541045

89. Klarenbach S, Cameron C, Singh S, Ur E (2011) Cost-effectiveness of second-line antihyperglycemic therapy in patients with type 2 diabetes mellitus inadequately controlled on metformin. Cmaj 183 (16):E1213-1220. doi:10.1503/cmaj.110178

90. Gao L, Zhao FL, Li SC (2012) Cost-utility analysis of liraglutide versus glimepiride as add-on to metformin in type 2 diabetes patients in China. Int J Technol Assess Health Care 28 (4):436-444. doi:10.1017/s0266462312000608

91. Nerat T, Locatelli I, Kos M (2016) Type 2 diabetes: cost-effectiveness of medication adherence and lifestyle interventions. Patient Prefer Adherence 10:2039-2049. doi:10.2147/ppa.S114602

92. Mostafa SA, Coleman RL, Agbaje OF, Gray AM, Holman RR, Bethel MA (2018) Modelling incremental benefits on complications rates when targeting lower HbA1c levels in people with Type 2 diabetes and cardiovascular disease. Diabet Med 35 (1):72-77. doi:10.1111/dme.13533

93. Pollock RF, Valentine WJ, Marso SP, Andersen A, Gundgaard J, Hallen N, Tutkunkardas D, Magnuson EA, Buse JB, Grp DS (2019) Long-term Cost-effectiveness of Insulin Degludec Versus Insulin Glargine U100 in the UK: Evidence from the Basal-bolus Subgroup of the DEVOTE Trial (DEVOTE 16). Applied Health Economics and Health Policy 17 (5):615-627. doi:10.1007/s40258-019-00494-3

94. Gray B, Schuetz CA, Weng W, Peskin B, Rosner B, Lipner RS (2012) Physicians' actions and influence, such as aggressive blood pressure control, greatly improve the health of diabetes patients. Health Affairs 31 (1):140-149. doi:10.1377/hlthaff.2011.0895

95. Dziuba J, Alperin P, Racketa J, Iloeje U, Goswami D, Hardy E, Perlstein I, Grossman HL, Cohen M (2014) Modeling effects of SGLT-2 inhibitor dapagliflozin treatment versus standard diabetes therapy on cardiovascular and microvascular outcomes. Diabetes Obes Metab 16 (7):628-635. doi:10.1111/dom.12261

96. Gaebler JA, Soto-Campos G, Alperin P, Cohen M, Blickensderfer A, Wintle M, Maggs D, Hoogwerf B, Han J, Pencek R, Peskin B (2012) Health and economic outcomes for exenatide once weekly, insulin, and pioglitazone therapies in the treatment of type 2 diabetes: a simulation analysis. Vasc Health Risk Manag 8:255-264. doi:10.2147/vhrm.S28744

97. Peskin BR, Shcheprov AV, Boye KS, Bruce S, Maggs DG, Gaebler JA (2011) Cardiovascular outcomes associated with a new once-weekly GLP-1 receptor agonist vs. traditional therapies for type 2 diabetes: a simulation analysis. Diabetes Obesity & Metabolism 13 (10):921-927. doi:10.1111/j.1463-1326.2011.01430.x

98. Cai X, Shi L, Yang W, Gu S, Chen Y, Nie L, Ji L (2019) Cost-effectiveness analysis of dapagliflozin treatment versus metformin treatment in Chinese population with type 2 diabetes. J Med Econ 22 (4):336-343. doi:10.1080/13696998.2019.1570220

99. Capel M, Ciudin A, Mareque M, Rodríguez RM, Oyagüez I (2018) COST-EFFECTIVENESS ANALYSIS OF EXENATIDE VERSUS GLP-1 RECEPTOR AGONISTS IN PATIENTS WITH TYPE 2 DIABETES MELLITUS. Value in Health 21:S129. doi:10.1016/j.jval.2018.09.773

100. Gu S, Wang X, Qiao Q, Gao W, Wang J, Dong H (2017) Cost-effectiveness of exenatide twice daily vs insulin glargine as add-on therapy to oral antidiabetic agents in patients with type 2 diabetes in China. Diabetes Obes Metab 19 (12):1688-1697. doi:10.1111/dom.12991

101. Tzanetakos C, Tentolouris N, Kourlaba G, Maniadakis N (2016) Cost-Effectiveness of Dapagliflozin as Add-On to Metformin for the Treatment of Type 2 Diabetes Mellitus in Greece. Clin Drug Investig 36 (8):649-659. doi:10.1007/s40261-016-0410-2

102. Tzanetakos C, Bargiota A, Kourlaba G, Maniadakis N (2016) Cost-effectiveness of exenatide once weekly versus insulin glargine and liraglutide for the treatment of type 2 diabetes in Greece. Value in Health 19 (7):A672

103. Gu S, Zeng Y, Yu D, Hu X, Dong H (2016) Cost-Effectiveness of Saxagliptin versus Acarbose as Second-Line Therapy in Type 2 Diabetes in China. PLoS One 11 (11):e0167190. doi:10.1371/journal.pone.0167190

104. Gu S, Mu Y, Zhai S, Zeng Y, Zhen X, Dong H (2016) Cost-Effectiveness of Dapagliflozin versus Acarbose as a Monotherapy in Type 2 Diabetes in China. PLoS One 11 (11):e0165629. doi:10.1371/journal.pone.0165629

105. Gordon J, McEwan P, Sabale U, Kartman B, Wolffenbuttel BH (2016) The cost-effectiveness of exenatide twice daily (BID) vs insulin lispro three times daily (TID) as add-on therapy to titrated insulin glargine in patients with type 2 diabetes. J Med Econ 19 (12):1167-1174. doi:10.1080/13696998.2016.1208207

106. Chuang LH, Verheggen BG, Charokopou M, Gibson D, Grandy S, Kartman B (2016) Cost-effectiveness analysis of exenatide once-weekly versus dulaglutide, liraglutide, and lixisenatide for the treatment of type 2 diabetes mellitus: an analysis from the UK NHS perspective. J Med Econ 19 (12):1127-1134. doi:10.1080/13696998.2016.1203329

107. Sabale U, Ekman M, Granström O, Bergenheim K, McEwan P (2015) Cost-effectiveness of dapagliflozin (Forxiga®) added to metformin compared with sulfonylurea added to metformin in type 2 diabetes in the Nordic countries. Primary Care Diabetes 9 (1):39-47. doi:10.1016/j.pcd.2014.04.007

108. Charokopou M, McEwan P, Lister S, Callan L, Bergenheim K, Tolley K, Postema R, Townsend R, Roudaut M (2015) Cost-effectiveness of dapagliflozin versus DPP-4 inhibitors as an add-on to Metformin in the Treatment of Type 2 Diabetes Mellitus from a UK Healthcare System Perspective. BMC Health Serv Res 15:496. doi:10.1186/s12913-015-1139-y

109. Deng J, Gu S, Shao H, Dong H, Zou D, Shi L (2015) Cost-effectiveness analysis of exenatide twice daily (BID) vs insulin glargine once daily (QD) as add-on therapy in Chinese patients with Type 2 diabetes mellitus inadequately controlled by oral therapies. J Med Econ 18 (11):974-989. doi:10.3111/13696998.2015.1067622

110. Charokopou M, McEwan P, Lister S, Callan L, Bergenheim K, Tolley K, Postema R, Townsend R, Roudaut M (2015) The cost-effectiveness of dapagliflozin versus sulfonylurea as an add-on to metformin in the treatment of Type 2 diabetes mellitus. Diabet Med 32 (7):890-898. doi:10.1111/dme.12772

111. van Haalen HG, Pompen M, Bergenheim K, McEwan P, Townsend R, Roudaut M (2014) Cost effectiveness of adding dapagliflozin to insulin for the treatment of type 2 diabetes mellitus in the Netherlands. Clin Drug Investig 34 (2):135-146. doi:10.1007/s40261-013-0155-0

112. Grzeszczak W, Czupryniak L, Kolasa K, Sciborski C, Lomon ID, McEwan P (2012) The cost-effectiveness of saxagliptin versus NPH insulin when used in combination with other oral antidiabetes agents in the treatment of type 2 diabetes mellitus in Poland. Diabetes Technol Ther 14 (1):65-73. doi:10.1089/dia.2011.0092

113. Erhardt W, Bergenheim K, Duprat-Lomon I, McEwan P (2012) Cost effectiveness of saxagliptin and metformin versus sulfonylurea and metformin in the treatment of type 2 diabetes mellitus in Germany: a Cardiff diabetes model analysis. Clin Drug Investig 32 (3):189-202. doi:10.2165/11597060-000000000-00000

114. Hou XY, Wan X, Wu B (2019) Cost-Effectiveness of Canagliflozin Versus Dapagliflozin Added to Metformin in Patients With Type 2 Diabetes in China. Frontiers in Pharmacology 10. doi:10.3389/fphar.2019.00480

115. Gupta V, Willis M, Johansen P, Nilsson A, Shah M, Mane A, Neslusan C (2019) Long-Term Clinical Benefits of Canagliflozin 100 mg versus Sulfonylurea in Patients with Type 2 Diabetes Mellitus Inadequately Controlled with Metformin in India. Value in Health Regional Issues 18:65-73. doi:10.1016/j.vhri.2018.06.002

116. Neslusan C, Teschemaker A, Willis M, Johansen P, Vo L (2018) Cost-Effectiveness Analysis of Canagliflozin 300 mg Versus Dapagliflozin 10 mg Added to Metformin in Patients with Type 2 Diabetes in the United States. Diabetes Ther 9 (2):565-581. doi:10.1007/s13300-018-0371-y

117. Johansen P, Hakan-Bloch J, Liu AR, Bech PG, Persson S, Leiter LA (2019) Cost Effectiveness of Once-Weekly Semaglutide Versus Once-Weekly Dulaglutide in the Treatment of Type 2 Diabetes in Canada. Pharmacoecon Open. doi:10.1007/s41669-019-0131-6

118. Ericsson A, Glah D, Lorenzi M, Jansen JP, Fridhammar A (2018) Cost-effectiveness of liraglutide versus lixisenatide as add-on therapies to basal insulin in type 2 diabetes. PLoS One 13 (2):e0191953. doi:10.1371/journal.pone.0191953

119. Steen Carlsson K, Persson U (2014) Cost-effectiveness of add-on treatments to metformin in a Swedish setting: liraglutide vs sulphonylurea or sitagplitin. J Med Econ 17 (9):658-669. doi:10.3111/13696998.2014.933110

120. Kuo S, Ye W, Duong J, Herman WH (2018) Are the favorable cardiovascular outcomes of empagliflozin treatment explained by its effects on multiple cardiometabolic risk factors? A simulation of the results of the EMPA-REG OUTCOME trial. Diabetes Research and Clinical Practice 141:181-189. doi:10.1016/j.diabres.2018.04.040

121. Herman WH, Ye W, Griffin SJ, Simmons RK, Davies MJ, Khunti K, Rutten GE, Sandbaek A, Lauritzen T, Borch-Johnsen K, Brown MB, Wareham NJ (2015) Early Detection and Treatment of Type 2 Diabetes Reduce Cardiovascular Morbidity and Mortality: A Simulation of the Results of the Anglo-Danish-Dutch Study of Intensive Treatment in People With Screen-Detected Diabetes in Primary Care (ADDITION-Europe). Diabetes Care 38 (8):1449-1455. doi:10.2337/dc14-2459

122. Ryabov I (2014) Cost-effectiveness of community health workers in controlling diabetes epidemic on the U.S.-Mexico border. Public Health 128 (7):636-642. doi:10.1016/j.puhe.2014.05.002

123. Shao H, Lin J, Zhuo X, Rolka DB, Gregg EW, Zhang P (2019) Influence of diabetes complications on HbA1c treatment goals among older U.S. adults: A cost-effectiveness analysis. Diabetes Care 42 (11):2136-2142. doi:10.2337/dc19-0381

124. Eastman RC, Javitt JC, Herman WH, Dasbach EJ, Copley-Merriman C, Maier W, Dong F, Manninen D, Zbrozek AS, Kotsanos J, Garfield SA, Harris M (1997) Model of complications of NIDDM. II. Analysis of the health benefits and cost-effectiveness of treating NIDDM with the goal of normoglycemia. Diabetes Care 20 (5):735-744

125. Gillett M, Brennan A, Watson P, Khunti K, Davies M, Mostafa S, Gray LJ (2015) The cost-effectiveness of testing strategies for type 2 diabetes: a modelling study. Health Technol Assess 19 (33):1-80. doi:10.3310/hta19330

126. O'Reilly D, Holbrook A, Blackhouse G, Troyan S, Goeree R (2012) Cost-effectiveness of a shared computerized decision support system for diabetes linked to electronic medical records. J Am Med Inform Assoc 19 (3):341-345. doi:10.1136/amiajnl-2011-000371
